# Preparation of soluble and functional recombinant HTLV-1 Tax protein using bacterial chaperones

**DOI:** 10.1242/bio.062659

**Published:** 2026-07-03

**Authors:** Michi Miura, Kazutaka Murayama, Satoshi Uemura, Hiroshi Kitamura, Mineki Saito, Teru Kanda

**Affiliations:** ^1^ Division of Microbiology, Faculty of Medicine, Tohoku Medical and Pharmaceutical University, Sendai 983-8536, Japan; ^2^ Division of Biomedical Measurements and Diagnostics, Graduate School of Biomedical Engineering, Tohoku University, Sendai 980-8579, Japan; ^3^ Division of Medical Biochemistry, Faculty of Medicine, Tohoku Medical and Pharmaceutical University, Sendai 983-8536, Japan; ^4^ Department of Microbiology, Kawasaki Medical School, Kurashiki 701-0192, Japan

**Keywords:** HTLV-1, Tax, Bacterial chaperones, Intrinsically disordered protein, Protein preparation, Protein structure

## Abstract

HTLV-1 viral protein Tax physically interacts with multiple host proteins to disrupt their normal functions, causing debilitating haematological malignancy and inflammatory disorders. The recombinant Tax protein, like other intrinsically disordered proteins, is insoluble when expressed in *Escherichia coli* due to the inclusion body formation, hampering structural studies of this important viral protein. We report that the bacterial chaperone Trigger Factor significantly enhances the solubility of HTLV-1 Tax fused to maltose-binding protein (MBP) as a solubility tag. Using a hexahistidine tag in addition to MBP enabled a two-step purification to isolate Tax from the MBP moiety after cleaving the fusion protein with HRV 3C protease. The purified recombinant protein activated NF-κB, indicating that the recombinant protein, solubilised by Trigger Factor, was functional. A molecular thermodynamics simulation, using a 3D model generated by AlphaFold2 as the initial state, suggested two putative domains within the Tax protein. These predicted domains could also be expressed and purified in the presence of exogenous Trigger Factor. By enabling the production of full-length and predicted domains of HTLV-1 Tax in the soluble form, this study takes the first step towards structural studies to understand how HTLV-1 Tax mechanistically interacts with various host factors.

## Teaser

**Summary:** Bacterial chaperone Trigger Factor opens up the structural analysis of HTLV-1 Tax by enabling the preparation of soluble and functional recombinant HTLV-1 Tax.

## INTRODUCTION

Recent advances in protein science are uncovering the prevalence of intrinsically disordered proteins and their links to human diseases. Approximately one-third of the human proteome is intrinsically disordered (Forman-Kay and Mittag, 2013), for which the traditional view of protein–protein interactions, often referred to as the key-and-keyhole model, does not apply. These proteins interact with a wide variety of other proteins via a long stretch of charged amino acids or pi-orbital electrons of aromatic amino acids (Borgia et al., 2018). Viruses with small genomes and limited coding capacity may benefit from such proteins, as a single protein can perform multiple functions, facilitating more complex interactions and enabling them to thrive in the host. The viral protein Tax, encoded by human T-cell leukaemia virus type 1 (HTLV-1), exemplifies this strategy.

HTLV-1 is the first retrovirus that was discovered to be pathogenic in humans. Approximately 10-20 million people worldwide are infected with this virus (Gessain and Cassar, 2012). HTLV-1 primarily infects CD4^+^ T cells and inserts a double-stranded copy of the viral genome into the host chromatin, where the integrated provirus persists in lymphocytes through mitotic cell division. After a long asymptomatic period, HTLV-1 causes adult T-cell leukaemia and inflammatory disorders in some infected individuals (Bangham et al., 2019).

Within its 9 kb genome, HTLV-1 encodes a limited number of accessory proteins by alternative splicing to persist in the host. HTLV-1 Tax, originally referred to as p40 (Sugamura et al., 1984), is one of these viral proteins comprising 353 amino acids and is responsible for viral pathogenesis. Shortly after the discovery of HTLV-1 and the Tax protein in the 1980s, many host proteins were identified as targets of Tax. Tax has multiple roles in pathogenicity. Among these, CREB and nuclear factor κB (NF-κB) molecules are likely the principal contributors to HTLV-1-associated diseases. Tax recruits the histone acetyltransferase p300 to CREB at the viral promoter (Kwok et al., 1996), thereby activating viral transcription. Tax activates the NF-κB signalling pathway by interacting with the NF-κB Essential Modulator (NEMO) (Harhaj and Sun, 1999; Yamaoka et al., 1998) to prevent apoptosis of infected cells. Tax also binds to the RNA helicase UPF1, thereby inhibiting its function (Fiorini et al., 2018). Although Tax has no known enzymatic activities, it enhances or inhibits the activity of a wide range of host factors by binding to their specific target proteins, thereby disrupting normal cellular functions. A recent study using biotin-proximity labelling identified more than 1500 host proteins targeted by Tax (Liang et al., 2025).

The cytosol and nucleosol are densely packed with proteins. If HTLV-1 Tax is highly adhesive to numerous host proteins in a promiscuous manner, Tax would form protein aggregates in the cell when it is abundantly produced in a transcription burst, in which hundreds to thousands of viral mRNAs are rapidly transcribed within a few hours (Billman et al., 2017; Miura et al., 2019). Therefore, we speculate that the interactions between HTLV-1 Tax and host proteins are rather specific: Tax undergoes conformational changes to fit its shape to the host protein it binds.

While extensive efforts have been made to identify the binding partners of HTLV-1 Tax in infected cells and their biological significance, the mechanism by which Tax interacts with various host proteins remains unknown. To date, no structural studies have been performed for Tax, presumably because Tax is insoluble, like many other intrinsically disordered proteins, when it is expressed in bacteria. In fact, as we set out to study the structure of HTLV-1 Tax to test our hypothesis, we realised that the soluble protein preparation was the first obstacle to overcome. Here, we report that co-expression of exogenous bacterial chaperones solubilises recombinant Tax proteins in *Escherichia coli*, thereby enabling the preparation of Tax in a soluble form. Our approach will facilitate structural analyses of Tax and its protein complexes with host factors by enabling the preparation of soluble recombinant proteins.

## RESULTS

### Tax is inherently insoluble when expressed in *E. coli*

**Fig. 1.**
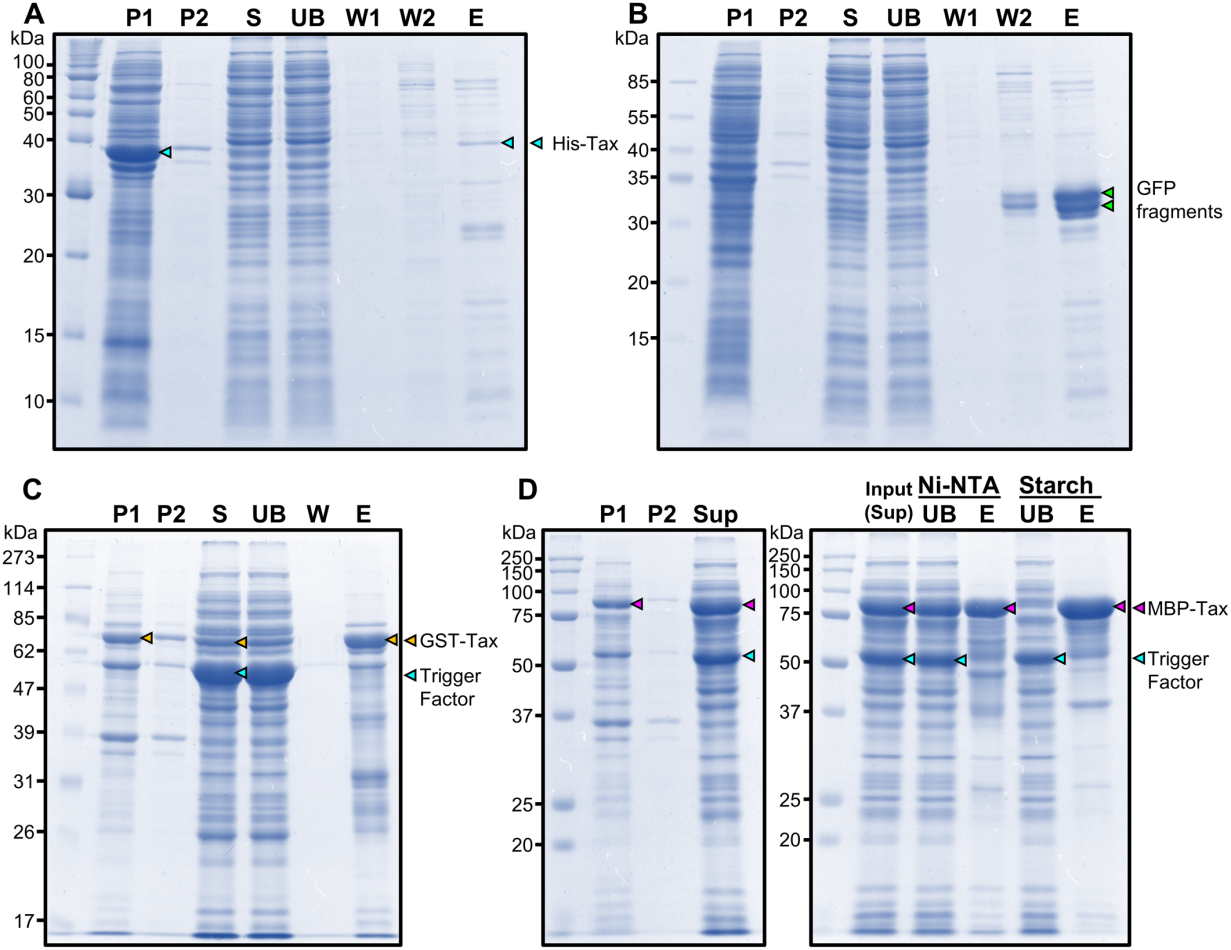
**Expression and purification of recombinant Tax proteins.** (A) Histidine-tagged HTLV-1 Tax expressed in BL21 (DE3) under the T7 promoter. During purification, proteins were fractionated and analysed by SDS-PAGE. Lanes P1, 2100×***g*** pellet; P2, 16,000×***g*** pellet; S, supernatant collected after 16,000×***g*** centrifugation; UB, unbound fraction from the Ni-NTA resin; W1, first wash; W2, second wash with 20 mM imidazole; E, elution with 400 mM imidazole. The arrowhead indicates the protein band corresponding to the expressed recombinant protein. (B) GFP-fused HTLV-1 Tax expressed in BL21 (DE3) under the T7 promoter. The lanes are labelled in the same way as in panel A. The arrowhead points to the major bands detected in lane E to guide the eye. (C) Purification of GST-fused Tax expressed from the pCold vector, together with Trigger Factor. Lanes P1, 2100×***g*** pellet; P2, 16,000×***g*** pellet; S, supernatant collected after 16,000×***g*** centrifugation; UB, unbound fraction from Ni-NTA; W, 20 mM imidazole wash; E, elution with 400 mM imidazole. The yellow arrowhead indicates GST-fused Tax, and the blue indicates Trigger Factor. (D) MBP-fused Tax expressed from the pCold vector, together with Trigger Factor. Lanes P1, 2100×***g*** pellet; P2, 16,000×***g*** pellet; Sup, supernatant collected after 16,000×***g*** centrifugation. The supernatant was subjected to purification using either Ni-NTA (lane Ni-NTA) or gelatinised corn starch (lane Starch). Lanes UB, unbound fraction; E, elution. The magenta arrowhead indicates MBP-fused Tax, and the blue is for Trigger Factor. (A-D) Each gel represents at least two independent purifications.

While bacteria have been used to express histidine-tagged recombinant Tax proteins for biochemical studies (Giebler et al., 1997; McCabe et al., 2013; Zhao and Giam, 1991), a recent study explicitly noted that Tax was insoluble in bacteria due to inclusion body formation (Fiorini et al., 2018), and this was also the case in our hands. We first tested a standard histidine-tagged recombinant protein purification, where a hexahistidine tag was attached to the N-terminus of Tax in a pET vector, and the recombinant protein was expressed in the *E. coli* strain BL21 (DE3) under the T7 promoter. The Tax protein (40 kDa) precipitated when the bacterial lysate was cleared by centrifugation (Fig. 1A, arrowhead in lane P1), and only a trace amount of the recombinant protein was recovered by Ni-NTA purification (Fig. 1A, arrowhead in lane E), indicating that the histidine-tagged Tax was insoluble.

When Tax was fused to GFP in the pET vector to visually track the recombinant protein (68 kDa) during purification, the final eluate from the Ni-NTA was bright green, indicating successful expression and purification; however, the molecular weight of the purified protein, estimated by SDS-PAGE, was about 32 kDa (Fig. 1B, arrowheads in lane E), consistent with the size of GFP. A western blot using an anti-His antibody confirmed that these major fragments detected by CBB staining were histidine-tagged N-terminal GFP, indicating that Tax in the C-terminal half of the recombinant protein was degraded (Fig. S1). An *in vitro* protein synthesis using the same plasmid produced the full-length GFP-Tax (Fig. S2), confirming the integrity of the constructed plasmid. These results suggest that Tax was unstable and degraded in bacteria, leaving only the GFP moiety intact.

Using a wheat germ cell-free protein expression system (Takai et al., 2010) for a quick assessment, we found that GST and maltose-binding protein (MBP) were promising solubility tags for Tax (Fig. S3). Thus, we attempted to express GST- and MBP-fused Tax using the pCold vector system (Qing et al., 2004) at a low temperature. Protein production at low temperatures using the pCold vector minimises protein degradation, aggregation, and inclusion body formation in bacteria and reduces the expression of bacterial housekeeping proteins while maintaining high production of recombinant protein under the control of the cold-shock promoter (Qing et al., 2004). Yet despite using the pCold vector system, most of the GST-fused Tax protein still precipitated in the pellet during the initial centrifugation to clear the bacterial lysate (Fig. S4). This result prompted us to use exogenous bacterial chaperones expressed alongside the recombinant Tax protein.

### Bacterial chaperones enhance the solubility of Tax

Trigger Factor is a bacterial chaperone that assists protein folding at the exit of the ribosome complex (Kramer et al., 2002). We used the *E. coli* strain BL21, carrying an additional plasmid encoding Trigger Factor, to express GST-fused Tax from the pCold vector along with the bacterial chaperone. Co-expression of Trigger Factor slightly improved the solubility of the GST-fused recombinant Tax (70 kDa), indicated by increased recovery of the recombinant protein in the soluble fraction (Fig. 1C, yellow in lane S). The recombinant protein was detected by SDS-PAGE as a major band in the Ni-NTA eluate (Fig. 1C, yellow in lane E).

The impact of Trigger Factor was most pronounced when GST was replaced with MBP, resulting in enhanced expression and solubility of recombinant Tax when co-expressed with Trigger Factor (Fig. 1D). The majority of the total MBP-fused Tax (86 kDa) was recovered in the soluble supernatant after centrifugation to clear the lysate (Fig. 1D, magenta in lanes P1 and Sup). We tested purification using either the Ni-NTA resin for the histidine tag attached to the N-terminus of MBP or gelatinised corn starch slurry (Kobashigawa et al., 2021) that captures the MBP moiety. Both methods enabled us to purify MBP-fused Tax as the single dominant protein in the eluate (Fig. 1D, magenta in lanes E of Ni-NTA and Starch), indicating that these purification tags can be efficiently used for a two-step purification to separate Tax from the MBP moiety, as described in the subsequent section. Notably, the gelatinised corn starch slurry achieved nearly complete recovery of the MBP-fused Tax from the cleared lysate, as indicated by the absence of MBP-Tax in the unbound fraction (Fig. 1D, lane UB of Starch).

Because of its excellent solubility and efficient purification of recombinant Tax as the single dominant protein with minimal co-purification of bacterial proteins, we proceeded with MBP-fused Tax in combination with Trigger Factor alone in our subsequent experiments.

### Separation of Tax from the solubility tag

**Fig. 2.**
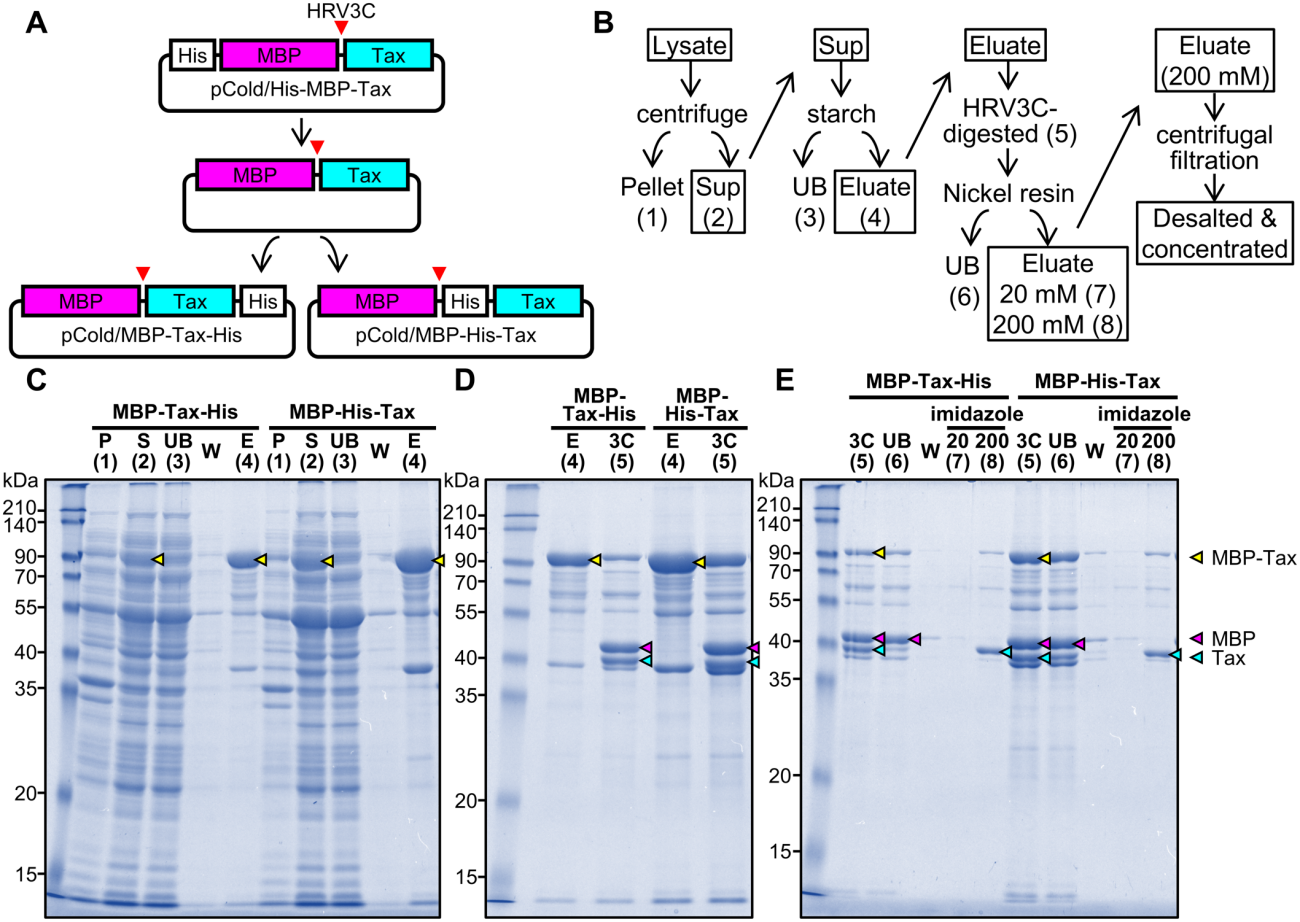
**Two-step purification to isolate Tax from the MBP moiety.** (A) Schematic diagram of the plasmid construction for MBP-fused Tax. (B) Overview of the two-step purification. The numbers associated with each fraction correspond to the lanes in the subsequent panels C-E. (C) First purification of the recombinant protein using gelatinised corn starch. Lanes P, 2100×***g*** pellet; S, supernatant after 16,000×***g*** centrifugation; UB, unbound from the gelatinised starch; W, wash; E, elution. The lanes for MBP-Tax-His and MBP-His-Tax are indicated above. The yellow arrowhead indicates MBP-fused Tax. (D) Digestion of MBP-fused Tax using HRV 3C protease. Lanes E, elution (the same as lane E in panel C); 3C, treated with HRV 3C. The yellow arrowhead indicates MBP-fused Tax, magenta is for cleaved MBP, and blue is for Tax. (E) Ni-NTA purification to isolate Tax. Lanes 3C, treated with HRV 3C (the same as lane 3C in panel D); UB, Ni-NTA unbound; W, wash with no imidazole; 20, 20 mM imidazole wash; 200, 200 mM imidazole elution. The yellow arrowhead indicates MBP-fused Tax, magenta is for cleaved MBP, and cyan is for Tax. (C-E) Each gel represents at least two independent purifications.

The original MBP-fused Tax in the pCold vector (Fig. 2A, pCold/His-MBP-Tax) contained an HRV 3C protease recognition site between MBP and Tax. We wished to separate Tax from the MBP moiety by cleaving the fusion protein with HRV 3C protease and subsequently isolating Tax with an additional affinity tag. To do this, the histidine tag was relocated from the N-terminus of the MBP moiety to the C-terminus of Tax on the plasmid through two rounds of site-directed mutagenesis (Fig. 2A, pCold/MBP-Tax-His). We also tested the N-terminal histidine tag for Tax purification by inserting a hexahistidine tag between the HRV 3C cleavage site and the N-terminus of Tax (Fig. 2A, pCold/MBP-His-Tax). This arrangement exposes the histidine tag at the N-terminus of Tax upon the cleavage of the fusion protein with HRV 3C protease.

Two-step purification was established to isolate Tax from the MBP solubility tag (Fig. 2B). First, the expressed recombinant proteins were recovered in the supernatant after the bacterial lysate was cleared (Fig. 2C, lane S), with the excellent solubility that is consistent with the original His-MBP-Tax (Fig. 1D). Next, we isolated the recombinant protein using the gelatinised starch for the MBP moiety. A single dominant band was detected in the eluate, corresponding to the MBP-fused Tax protein (Fig. 2C, arrowhead in lane E). The eluted protein was then treated with the HRV 3C protease. SDS-PAGE resolved two protein bands with molecular weights consistent with those of MBP (45 kDa) and Tax (41 kDa) (Fig. 2D, magenta and cyan in lane 3C, respectively), confirming the efficient cleavage of the fusion protein. This result indicates that the HRV 3C protease recognition site was accessible, free from steric hindrance by potentially unstructured Tax, and that the enzyme could access the recognition site in MBP-His-Tax that is in proximity to the positive charge of the hexahistidine tag. Finally, Tax was isolated via the hexahistidine tag at its C- or N-terminus and eluted with 200 mM imidazole (Fig. 2E, cyan in lane 200).

### The recombinant Tax folded by Trigger Factor activates NF-κB

**Fig. 3.**
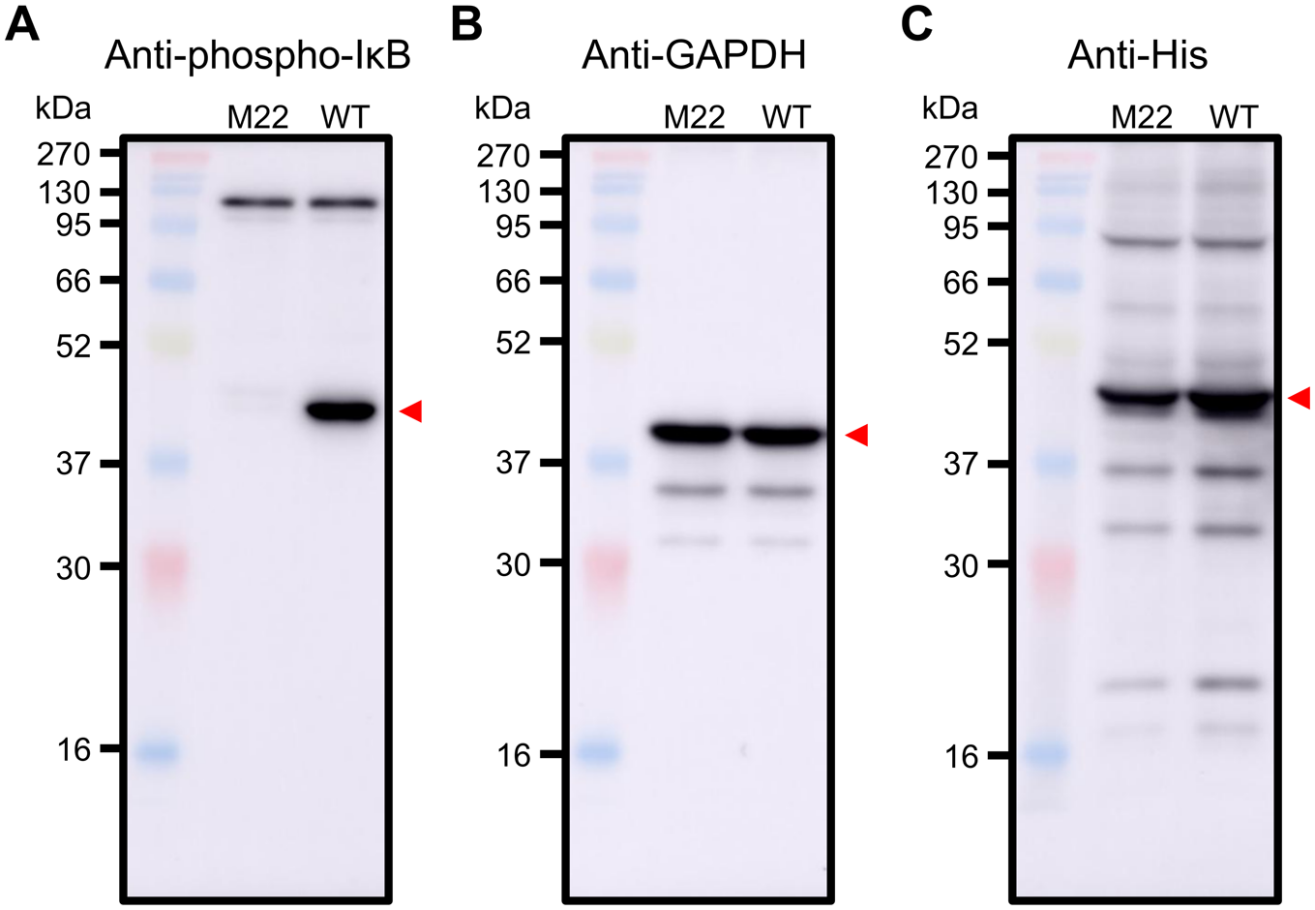
**Activation of NF-κB by the recombinant Tax protein folded by Trigger Factor.** (A) Western blot to detect phosphorylated IκB. The reaction mixtures, each containing Jurkat cell extract and the test protein (either WT or M22, as indicated), were analysed. The red arrowhead indicates the band of phosphorylated IκB, identified by its molecular size; the faint bands between 95 and 130 kDa are nonspecific. (B) Western blot to detect GAPDH (arrowhead) from the Jurkat cell extract in the reaction mixtures. (C) Western blot to detect the histidine-tagged recombinant proteins (arrowhead) in the reaction mixtures. (A-C) The blots are representative of two independent experiments using separate protein preparations.

We wished to confirm whether the recombinant Tax, solubilised with the bacterial chaperone Trigger Factor, is functional using an *in vitro* NF-κB assay (Shibata et al., 2011). This assay uses mammalian cell extract and exogenous ATP to determine whether the test protein activates the NF-κB pathway by detecting the phosphorylation of the endogenous inhibitor of κB (IκB) in the cell extract. Addition of the purified recombinant Tax (Fig. 2E, lane 200 of MBP-Tax-His) induced IκB phosphorylation (Fig. 3A, lane WT). As a negative control, a Tax mutant M22, defective in NF-κB activation (Smith and Greene, 1990), was generated by mutating the Tax-coding sequence on the expression plasmid and prepared alongside the wild-type Tax protein. In contrast to the wild-type Tax, the Tax mutant M22 failed to induce IκB phosphorylation (Fig. 3A, lane M22). We confirmed that equivalent amounts of cell lysate (Fig. 3B) and the respective test protein (Fig. 3C) were loaded in each reaction. These results exclude the possibility that the IκB phosphorylation was caused by contaminants derived from the bacterial lysates in the test protein preparation and indicate that the recombinant Tax, solubilised using Trigger Factor, retains the ability to activate NF-κB, a primary function of Tax in the development of HTLV-1-related diseases.

### *In silico* analysis of HTLV-1 Tax

**Fig. 4.**
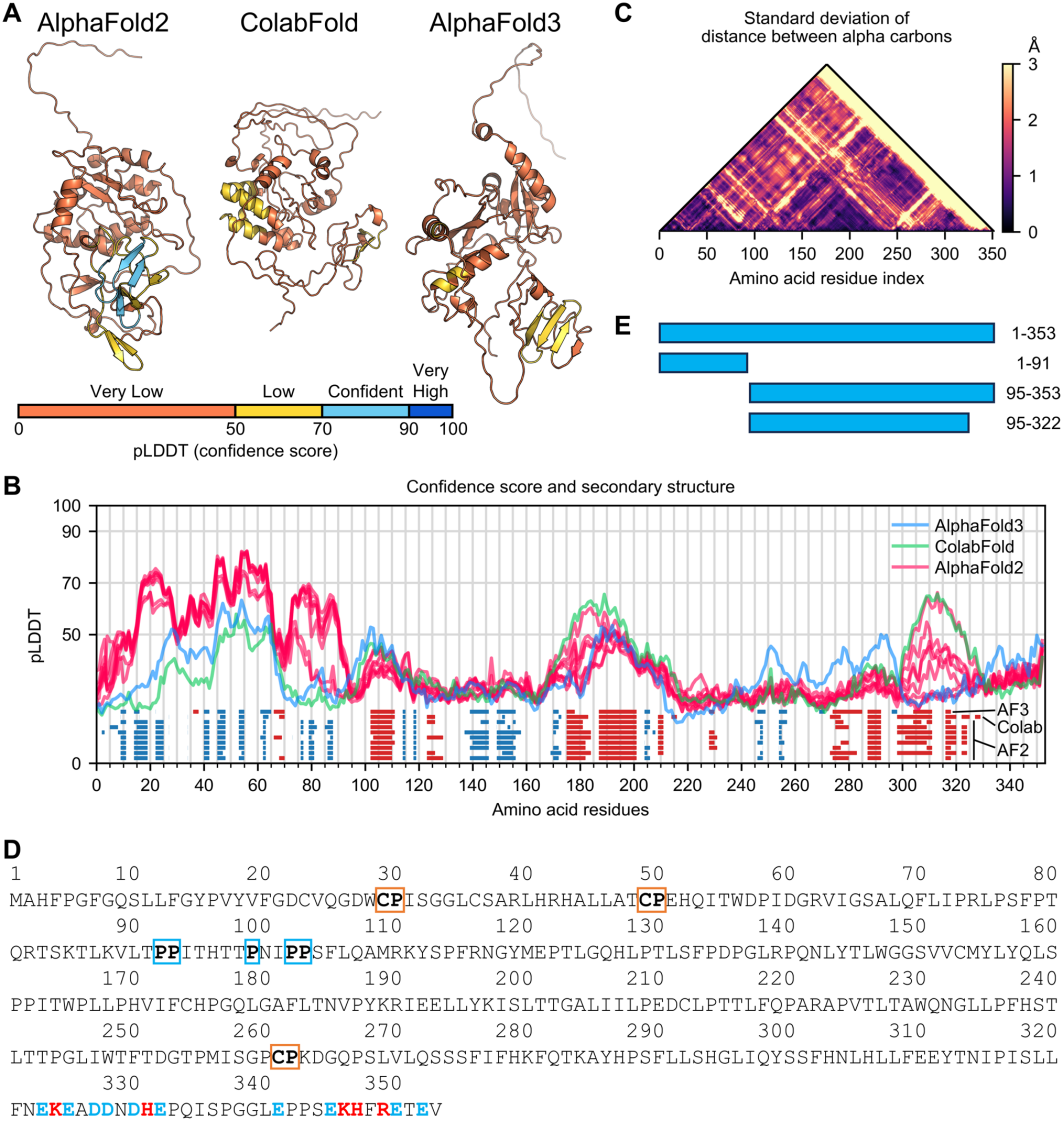
***In silico* analyses of HTLV-1 Tax.** (A) AlphaFold prediction of HTLV-1 Tax. The results for three variants (AlphaFold2, ColabFold, and AlphaFold3) are presented with a colour code indicating the confidence score. (B) The confidence score and the predicted secondary structure are plotted along the amino acid sequence. The red horizontal bars indicate regions predicted as α-helices, and the blue bars indicate β-sheets. The eight lines of horizontal bars at the bottom for the secondary structure prediction are from AlphaFold2, and the other two lines are from ColabFold and AlphaFold3, as indicated in the plot. (C) Heat map showing the standard deviation of distance between amino acids (alpha carbons), illustrating the fluctuations of amino acids in relation to other residues in the Tax protein during the thermodynamics simulation. (D) The amino acid sequence of Tax. The proline residues around amino acid position 100 are indicated with blue rectangles; charged amino acids after position 321 are coloured blue (positive charge) and red (negative charge); and the CP motif is highlighted with orange rectangles. (E) The truncated Tax fragments were designed according to the domain prediction shown in panel C. The length of the bars (i.e. the amino acid positions) corresponds to the horizontal axis of the heat map in panel C to highlight the relationship between the fragments and the predicted domains.

Having confirmed that the preparation of soluble and functional recombinant HTLV-1 Tax is feasible, we wish to gain insights into its putative structure. AlphaFold and its variants have emerged as valuable tools for predicting 3D protein structures, especially when experimentally determined structures are unavailable. We used AlphaFold2 (Jumper et al., 2021), ColabFold (Mirdita et al., 2022) and AlphaFold3 (Abramson et al., 2024) to predict the protein structure of HTLV-1 Tax (Fig. 4A). These methods produced similar results, with some discernible differences: α-helices were consistently predicted in regions such as amino acids 102-110 and 187-201 (Fig. 4B), whereas some β-sheets within the first 95 amino acids were predicted only by AlphaFold2 with high confidence (Fig. 4B).

We wished to exploit the AlphaFold prediction to infer the putative domain architecture of the Tax protein. To this end, we performed molecular dynamics simulations to investigate the thermodynamic fluctuations of the Tax molecule using one of the eight AlphaFold2 predictions (Fig. S5) as the initial state. If Tax is composed of certain protein domains, these domains would be relatively stable, and the residues within the same domains would maintain constant distances from each other during the thermodynamic fluctuation. By plotting the standard deviations of distances between all pairs of amino acids throughout the simulation, we identified putative domains spanning residues 1-95, 100-240, and 250-320 (Fig. 4C). Notably, two consecutive proline residues at positions 92 and 93 and at positions 102 and 103, flanking another proline at position 99 (Fig. 4D, blue rectangles), appeared to mark the boundary of these putative domains. The rest of the C-terminal region, amino acids 321-353, appeared to be disordered, as the standard deviation of distances to other residues was high (Fig. 4C), indicating that this region fluctuated in an unstructured manner. This region contains a stretch of charged amino acids (Fig. 4D, cyan and red) and is predicted to be disordered by AIUPred (Erdos and Dosztanyi, 2024) and PONDR VSL2 (Peng et al., 2006) (Fig. S6).

### Preparation of predicted Tax domains

**Fig. 5.**
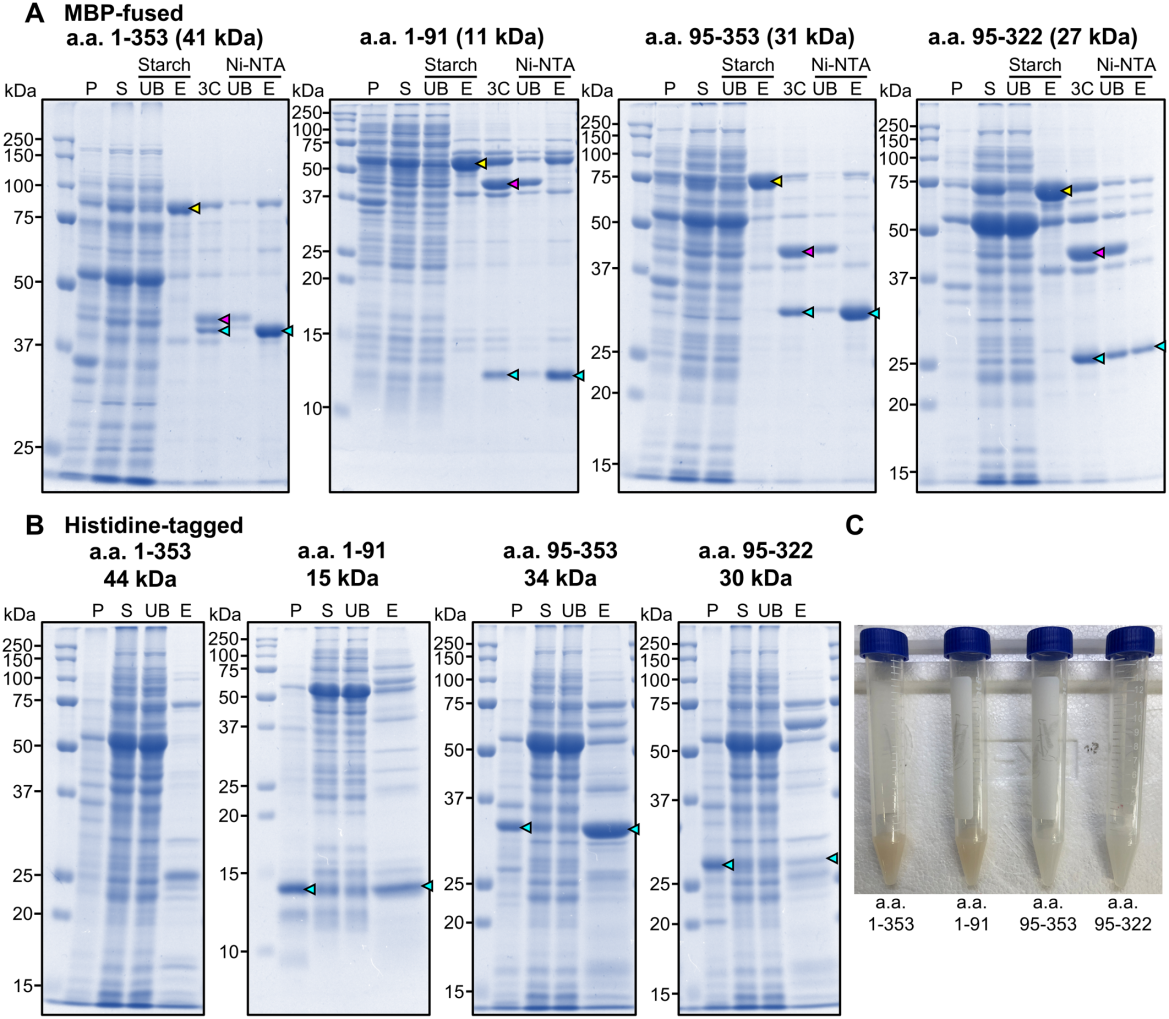
**Expression and purification of predicted Tax domains.** (A) MBP-fused full-length Tax (amino acids 1-353) and Tax fragments (amino acids 1-91, 95-353, and 95-322) were purified using gelatinised starch, treated with HRV 3C, and then Tax was isolated using Ni-NTA. Lanes P, 2100×***g*** pellet; S, supernatant after 16,000×***g*** centrifugation; Starch UB, unbound from the gelatinised starch; Starch E, elution; 3C, treated with HRV 3C; Ni-NTA UB, unbound from Ni-NTA; and Ni-NTA E, 400 mM imidazole elution. The yellow arrowhead indicates the MBP-fused fragment, magenta is for cleaved MBP, and blue is for the Tax fragment. The molecular weight of the Tax fragment that appears after the cleavage of MBP is indicated above each panel. (B) Expression and purification of histidine-tagged Tax fragments with the co-expression of exogenous Trigger Factor. The blue arrowhead indicates the expressed recombinant protein. The molecular weight of the histidine-tagged Tax fragment is indicated above each panel. (C) The colour change in gelatinised corn starch used for the purification of MBP-fused Tax protein. The photograph of the starch slurry was taken after the recombinant protein was bound and pelleted at the bottom of the tube. (A,B) Each gel represents at least two independent purifications.

We examined whether these predicted domains could also be expressed in a soluble form and separated from the MBP tag using HRV 3C. To this end, three truncated Tax fragments corresponding to amino acids 1-91, 95-353, and 95-322 (Fig. 4E) were designed. DNA fragments coding for these three regions were inserted into the pCold MBP vector to express MBP-fused Tax fragments (56, 76 and 72 kDa, respectively). Each of these three plasmids was modified to relocate the histidine tag to the C-terminus of the Tax fragment as described earlier. All these MBP-fused fragments were recovered in the soluble fraction and were successfully purified using the gelatinised starch (Fig. 5A, yellow in lane E of Starch). The Tax fragments were then separated from the MBP moiety following HRV 3C cleavage (Fig. 5A, cyan and magenta, respectively, in lane 3C). Although the Tax fragment spanning amino acids 95-322 showed inefficient binding to the Ni-NTA resin following the cleavage of MBP (Fig. 5A, lane UB of Ni-NTA), all the predicted Tax domains could be isolated from MBP and purified (Fig. 5A, cyan in lane E of Ni-NTA).

GFP-fused fragments of these putative Tax domains were also expressed from the pCold vector and successfully recovered from the lysate in the presence of exogenous Trigger Factor (Fig. S7A), in contrast to our earlier result of GFP-fused Tax where the Tax moiety was degraded in bacteria (Fig. 1B). These GFP-fused Tax fragments can be a useful tool to assess whether Tax forms, or is incorporated into, liquid droplets during liquid-liquid phase separation.

Since we observed the significant effect of Trigger Factor on enhancing the solubility of MBP-fused recombinant Tax, we asked whether Tax would be soluble on its own if Trigger Factor was co-expressed. To test this, we prepared histidine-tagged full-length Tax and the three putative Tax domains (Fig. 4E) in the pCold empty vector from which GST was removed. The results varied among the fragments (Fig. 5B). The full-length Tax showed no detectable protein expression. The Tax fragment spanning amino acids 95-322 yielded only a trace amount of protein in the eluate, possibly due to poor binding to the Ni-NTA resin as was observed earlier in the MBP-fused form (Fig. 5A). The other fragments tested, spanning amino acids 1-91 and 95-353, showed good protein yield, offering an alternative way to prepare the Tax fragments for functional and structural assays.

### Tax is potentially a heme-binding protein

Lastly, during the preparation of these Tax fragments, we noted that Tax exhibits a brownish colour. This observation was most evident when the corn starch slurry, bound with MBP-fused recombinant Tax, was pelleted by centrifugation (Fig. 5C) or when the bacteria were harvested after GFP-fused recombinant Tax was expressed (Fig. S7B). These colour changes were observed only for the full-length Tax or the predicted Tax domain spanning amino acids 1-91 and not for the predicted domains spanning amino acids 95-353 or 95-322. The first 90 amino acids of the Tax protein contain two cysteine-proline (CP) motifs (Fig. 4D, orange rectangles). The CP motif in a protein is known to bind heme (Watanabe-Matsui et al., 2015; Wissbrock et al., 2019). These observations raise the possibility that Tax binds heme primarily within the first 90 amino acids and that heme binding accounts for the brownish appearance of Tax.

## DISCUSSION

For structural studies to understand how HTLV-1 Tax functions mechanistically with various host proteins, preparation of soluble recombinant proteins is essential. In this study, we demonstrated that the bacterial chaperone Trigger Factor efficiently solubilises MBP-fused recombinant Tax proteins expressed in *E. coli*. The recombinant Tax protein, assisted by Trigger Factor, was functional in activating NF-κB. In addition to the protein preparation and biochemical characterisation, we identified putative domains within HTLV-1 Tax using AlphaFold predictions and subsequent molecular thermodynamics simulations. These predicted domains could also be expressed, cleaved from the solubility tag, and isolated in the soluble fraction.

In addition to Trigger Factor, we also tested other bacterial chaperones, GroEL/ES and DnaKJE (Fig. S8). GroEL and DnaJ were co-purified with GST-fused Tax, possibly because these bacterial chaperones have a high affinity for Tax or vice versa when expressed in bacteria. Therefore, using Trigger Factor alone was sufficient for solubilising Tax while minimising the co-purification of bacterial chaperones.

The effect of Trigger Factor was most pronounced when Tax was fused to MBP, and the recovery rate of the MBP-fused Tax protein was excellent using the corn starch slurry, thereby contributing to maximising the protein yield. Since preparing the gelatinised starch is inexpensive (Kobashigawa et al., 2021), we used large amounts (∼15% of the input volume) for purification. Although we did not systematically evaluate Ni-NTA purification efficiency by titrating the amount of resin used in this study, increasing the amount of Ni-NTA resin could potentially improve the recovery rate of the recombinant protein during Ni-NTA purification.

Tax is a difficult protein to solubilise, as described in our study and others (Fiorini et al., 2018), and is expected to be highly disordered. Therefore, Tax may not crystallise on its own. We speculate that Tax adopts a conformation that is specific to each binding partner. Cryogenic electron microscopy (cryo-EM) or nuclear magnetic resonance (NMR), in addition to spectroscopy and light-scattering techniques, would be promising approaches for structural analysis. However, each of these methods involves intricate technicalities to consider for the application to Tax. Using cryo-EM to analyse protein complexes formed by Tax and host factors, both prepared *in vitro*, would be appealing, though selecting appropriate binding partners for Tax to form a stable, uniform complex of approximately 100 kDa in size would be essential for success. NMR faces another technical limitation, as it favours smaller protein molecules for better signal resolution. The full-length Tax, with its size of 40 kDa, might be slightly too large for optimal NMR analysis.

Addressing these technical challenges, our study suggests at least three immediate future directions. First, we incidentally found that the bacterial chaperone GroEL co-purifies with Tax, indicating that GroEL and Tax form a protein complex with strong binding affinity. Although GroEL is not a natural binding partner in the mammalian host, it is worth analysing this molecular complex by cryo-EM to obtain the first 3D structure of Tax – GroEL is a bacterial homologue of a human heat shock protein, and the human heat shock protein family is known to be associated with Tax in the host cell and protect it from proteasomal degradation (Cheng et al., 2001; Gao and Harhaj, 2013). Second, we found that the Tax fragment comprising the first 91 amino acids can be expressed and purified even without a solubility tag at its N-terminus, making it a suitable candidate for NMR analysis. Interestingly, heme was suggested to bind to this region. Analysing this putative N-terminal domain of Tax as it binds heme in solution by NMR would be intriguing. It is also important to investigate the biological impact of heme binding to Tax on its functions in the cellular environment as well as its potential contribution to the HTLV-1-associated pathogenesis.

In this paper, we demonstrated the preparation of soluble, functional recombinant HTLV-1 Tax using bacterial chaperones. Additional purification, such as size-exclusion chromatography, is required to improve the purity of the recombinant protein for structural studies. Nevertheless, we believe that this work represents a first step towards the structural characterisation of HTLV-1 Tax.

## MATERIALS AND METHODS

### Plasmid construction

The Tax-coding fragment was obtained from an HTLV-1^+^ T-cell clone TBX4B (Satou et al., 2016), recently isolated and maintained *in vitro*. Total RNA was extracted, and cDNA was synthesised using an oligo dT primer. The Tax-coding fragment was amplified by PCR using the primers NHisTax-F2 and NHisTax-R (Table 1) and was inserted into pETBA (Biodynamics Laboratory, Japan) at the NdeI and NotI restriction sites. GFP-fused Tax in the pET vector was constructed by first inserting the GFP fragment, amplified from pEGFP-C1 (Clontech) using NHisGFP-F and NHisGFP-R (Table 1), into the pETBA vector at the NdeI and NotI sites and then inserting the Tax-coding fragment, amplified using TEV-tax-F and NHisTax-R (Table 1), downstream of the GFP at the NotI site.

**Table 1.** Primers used for plasmid construction

Name	Sequence (5′ to 3′)
NHisTax-F2	GGG CATATG CAT CAT CAT CAT CAT CAC ATG GCC CAC TTC CCA GG
NHisTax-R	GGG GCGGCCGC GAC TTC TGT TTC TCG GAA ATG TTT TTC
NHisGFP-F	GGG CATATG CAT CAT CAT CAT CAT CAT GTG AGC AAG GGC GAG GAG
NHisGFP-R	GGG GCGGCCGC CTT GTA CAG CTC GTC CAT GCC
TEV-tax-F	AA GCGGCCGC A GAA AAC CTG TAT TTT CAG GGC ATG GCC CAC TTC CCA GG
SalI-tax-F	GGG GTC GAC ATG GCC CAC TTC CCA GG
SalI-tax-R	GGG GTC GAC TCA GAC TTC TGT TTC TCG GAA ATG TTT TTC
SalI-TaxD1-R	GGG GTC GAC TCA GGT AAG GAC CTT GAG GGT CTT
SalI-TaxD2-F	GGG GTC GAC ACT CAT ACA ACC CCC AAC ATT C
SalI-TaxD2-R	GGG GTC GAC TCA GTT AAA AAG TAG AGA AAT GGG GAT GTT G
pColdGST2MBP-F	GCGGCCGC C CCT ACC TTC GAT ATG ATG ATG AT
pColdGST2MBP-R	ATT ACTAGT CTG GAA GTT CTG TTC CAG G
MBP-F	GGG GCGGCCGC GAA GAA GGT AAA CTG GTA ATC TGG
MBP-R	GGG ACTAGT GTT GTT GTT ATT GTT ATT GTT GTT GTT G
250807_RemHis-1	CAC TTT GTG ATT CAT GGT GTA TTA CC
250807_RemHis-2	ATC GAA GGT AGG GGC GG
250829	CAT CAT CAT CAT CAT CAT TGA GTC GAC CTG CAG TCT
250819-2	GAC TTC TGT TTC TCG GAA ATG TTT TTC
250819-3	GGT AAG GAC CTT GAG GGT CT
250819-4	GTT AAA AAG TAG AGA AAT GGG GAT GTT G
250912-1	ATG ATG ATG ATG GTG ATG CGG GCC CTG GAA CAG AA
250912-2	GGA CAT ATG GAG CTC GGT ACC
251210_NotI-EGFP-F	GGG GCGGCCG CAT GGT GAG CAA GGG CGA GGA G
251210_SpeI-EGFP-R	GGG ACTAGT CTT GTA CAG CTC GTC CAT GC
EcoRV-tax-F	GTT GATATC ATG GCC CAC TTC CCA GG
SpeI-tax-R	GGG ACTAGT TCA GAC TTC TGT TTC TCG GAA ATG TTT TTC

An overview of the plasmid construction using the pCold vector is illustrated in Fig. S9. First, pCold/His-GST-Tax (full-length and truncated fragments) (Fig. S9) was constructed by inserting the fragment into the original pCold GST DNA vector (Takara Bio) using the SalI restriction site. The Tax fragments were amplified using SalI-tax-F and SalI-tax-R (for full length), SalI-tax-F and SalI-TaxD1-R (for amino acids 1-91), SalI-TaxD2-F and SalI-tax-R (for amino acids 95-353), and SalI-TaxD2-F and SalI-TaxD2-R (for amino acids 95-322) (Table 1).

The original pCold GST DNA vector was modified to construct pCold/His-MBP-Tax (Fig. S9). First, the GST-coding region was removed by inverse PCR with primers pColdGST2MBP-F and pColdGST2MBP-R (Table 1) and subsequent ligation using a KOD Plus Mutagenesis Kit (Toyobo, SMK-101) to create the vector designated pCold (Fig. S9). The MBP-coding fragment, obtained from the pMAL-c6T vector (NEB) by PCR using primers MBP-F and MBP-R (Table 1), was then inserted in place of GST using the NotI and SpeI sites to create pCold MBP. Subsequently, the Tax-coding fragment from pCold/His-GST-Tax was subcloned downstream of MBP using KpnI and XbaI to establish pCold/His-MBP-Tax (Fig. S9). The histidine tag at the N-terminus of MBP was removed by inverse PCR with primers 250807_RemHis-1 and 250807_RemHis-2 (Table 1) and by subsequent ligation to create pCold/MBP-Tax. A hexahistidine tag was then inserted at either the C- or N-terminus of Tax by another round of mutagenesis to construct pCold/MBP-Tax-His or pCold/MBP-His-Tax, respectively (Fig. S9). To insert the hexahistidine tag at the C-terminus, primers 250829 and 250819-2 (for full length and amino acids 95-353), 250829 and 250819-3 (for amino acids 1-91), and 250829 and 250819-4 (for amino acids 95-322) (Table 1) were used for the inverse PCR. Primers 250912-1 and 250912-2 (Table 1) were used to insert the hexahistidine tag at the N-terminus of the full-length Tax.

pCold GFP was created by inserting the GFP fragment, amplified using primers 251210_NotI-EGFP-F and 251210_SpeI-EGFP-R (Table 1), into the NotI and SpeI sites in pCold (Fig. S9). The Tax-coding fragments from pCold/His-GST-Tax were subcloned downstream of GFP using KpnI and XbaI to establish pCold/His-GFP-Tax (Fig. S9). The Tax-coding fragments were also subcloned into pCold to create pCold/His-Tax (Fig. S9).

The expression plasmids for wheat-germ cell-free expression system were either constructed using the original pEU vector series (CellFree Sciences, Japan) or synthesised by Integrated DNA Technologies (IDT). pEU-GST-Tax for the expression of GST-fused Tax used in Fig. S3 was constructed by inserting the Tax-coding fragment, amplified using primers EcoRV-tax-F and SpeI-tax-F (Table 1), into the pEU vector pEU-E01-GST-PS-MCS-N1 (CellFree Sciences, Japan) at the EcoRV and SpeI sites. The other plasmids used in Fig. S3 for wheat-germ cell-free protein expression of SUMO-fused and MBP-fused Tax were synthesised by IDT with codons optimised for wheat-germ expression.

The sequences of all plasmids described above were verified by Sanger sequencing.

### Protein expression

Histidine-tagged Tax and GFP-fused Tax from pETBA (Fig. 1A,B) were induced with 0.1 mM isopropylthiogalactoside (IPTG) at 23°C overnight in BL21 (DE3) cultured in Luria Bertani (LB) broth containing 100 µg/ml ampicillin. The recombinant Tax protein from the pCold vector without exogenous bacterial chaperones was induced with 0.1 mM IPTG at 15°C overnight in BL21 (DE3) cultured in LB containing 100 µg/ml ampicillin.

For the co-expression of bacterial chaperones, BL21 competent cells carrying pTf16 (Takara Bio, 9125), pG-Tf2 (Takara Bio, 9124), or pG-KJE8 (Takara Bio, 9121) (Nishihara et al., 2000) were transformed with the pCold Tax-expression plasmid. The bacteria were grown in the presence of 100 µg/ml ampicillin and 25 µg/ml chloramphenicol. The bacterial chaperones were induced with 0.5 mg/ml arabinose (pTf16), 1 ng/ml tetracycline (pG-Tf2), or both 0.5 mg/ml arabinose and 1 ng/ml tetracycline (pG-KJE8), in addition to 0.1 mM IPTG for the Tax-protein induction, at 15°C overnight.

All protein expression was induced when the optical density of the bacterial culture at 600 nm reached around 0.6. After protein expression, bacteria were harvested by centrifugation and stored at −80°C until use.

### Protein extraction and purification

The frozen bacterial pellet was thawed in the lysis buffer comprising 50 mM Tris-HCl (pH 8.0), 400 mM NaCl, 10% glycerol, 0.1% Triton X-100, and 1 mM tris(2-carboxyethyl)phosphine (TCEP) supplemented with an EDTA-free protease inhibitor cocktail (Roche, 04693159001). The bacterial cell wall was disrupted on ice using an ultrasonic cell disruptor (Misonix, XL2000) for six cycles of 30 s on and 30 s off at an output power of 7 W. The bacterial lysate was cleared by centrifugation (2100×***g***, 10 min, and 4°C, followed by 16,000×***g***, 10 min, and 4°C).

For the batch purification using hexahistidine or GST, the supernatant was combined with Ni-NTA or glutathione resin and incubated at 4°C. The resin was collected by centrifugation, and the unbound fraction was separated. After the resin was washed, the protein was eluted with 200 mM (Fig. 2E) or otherwise 400 mM imidazole for histidine or 10 mM reduced L-glutathione (Sigma-Aldrich, G4251) for GST. For the purification using MBP, the supernatant was combined with the in-house gelatinised starch slurry (Kobashigawa et al., 2021) prepared from corn starch powder (Wako, 193-09925) and incubated at 4°C. The slurry was collected by centrifugation, and the unbound fraction was separated. After the slurry was washed, the protein was eluted with 90 mM maltose (Nacalai Tesque, 21116-92).

Protease digestion using HRV 3C (Nippon Gene, 317-09301) was performed at 4°C overnight with a protease concentration of ∼4 µg/ml.

For a step-by-step protocol for purifying MBP-fused Tax, please see https://www.protocols.io/view/purification-of-mbp-fused-htlv-1-tax-miura-et-al-2-kxygxj6n4l8j/v1.

### *In vitro* protein synthesis

The GFP-fused Tax was synthesised *in vitro* from the same plasmid used in Fig. 1B, alongside other control plasmids, using the PURExpress *In Vitro* Protein Synthesis Kit (NEB, E6800S) according to the manufacturer's instructions.

Wheat-germ protein expression was performed using a WEPRO7240 Core Kit (CellFree Sciences, CFS-C7). Namely, RNA was transcribed from 1 µg of the template plasmid at 37°C for 6 h using SP6 RNA polymerase. The transcription reaction was combined with an equal volume of wheat-germ extract and 40 µg/ml creatine kinase. The mixture was dispensed at the bottom of a 96-well plate, beneath 200 µl of an amino acid solution, to form a discrete sub-layer. The plate was then incubated at 15°C overnight.

### *In vitro* NF-κB activation assay

The assay was performed according to Shibata et al. (2011) with some modifications. Jurkat cell extract was prepared by homogenising ∼3×10^7^ cells in 100 µl of hypotonic buffer [10 mM Tris-HCl, pH 8.0, 1.5 mM MgCl_2_, 10 mM KCl, and 0.5 mM dithiothreitol (DTT)] in a 1.5 ml tube and clearing the debris by centrifugation (17,700×***g***, 10 min, and 4°C). The cell extract was combined with 2 mM ATP and ∼1 µM of test protein and incubated at 30°C for 1.5 h in the presence of phosphatase inhibitors (Nacalai Tesque, 07575-51). Phosphorylated IκB, glyceraldehyde 3-phosphate dehydrogenase (GAPDH), and the histidine-tagged recombinant Tax were detected by western blotting using mouse anti-phospho-IκBα monoclonal antibody (Cell Signaling Technology, 9246, 1:1000 dilution), rabbit anti-GAPDH monoclonal antibody (Cell Signaling Technology, 2118, 1:1000 dilution), and mouse anti-His-tag monoclonal antibody (Apro Science, AB-2001, 1:1000 dilution), respectively, with the horseradish peroxidase (HRP)-labelled anti-mouse IgG (Cytiva, NA931V, 1:2000, for phospho-IκB and His-tag) and anti-rabbit IgG (Cytiva, NA934V, 1:2000, for GAPDH). The signal was detected using ECL Prime Western Blotting Detection Reagent (Cytiva, RPN2232).

### AlphaFold prediction

AlphaFold2 was installed according to the instructions on github.com/google-deepmind/alphafold with some workarounds and run on the local computer. ColabFold was run on colab.research.google.com/github/deepmind/alphafold/blob/main/notebooks/AlphaFold.ipynb, and AlphaFold3 was run on alphafoldserver.com. A National Center for Biotechnology Information (NCBI) reference sequence for HTLV-1 Tax (NP_057864) was used for prediction. The resulting models in the PDB format were visualised using PyMOL version 3.0.0.

### Molecular dynamics simulation

OpenMM (Eastman et al., 2024) was used with the NVIDIA CUDA platform. The initial protein coordinates were obtained from a PDB model of HTLV-1 Tax, predicted by AlphaFold2 as described above. The AMBER14SB force field was used for protein atoms and the TIP3P-FB for water molecules. Sodium and chloride ions were included at 0.1 M in the system. Long-range electrostatic interactions were treated with the Particle Mesh Ewald method, using a 1.0 nm non-bonded cutoff. The system was energy-minimised, and then the simulation was performed for 100 ns in the NPT ensemble at 1.0 bar and 310 K with an integration time step of 2 fs. The protein coordinates were sampled every 10,000 steps (5000 time points over 100 ns).

### IDR prediction

Intrinsically disordered regions were predicted using AIUPred v2 (aiupred.elte.hu) (Erdos and Dosztanyi, 2024) and PONDR VSL2 (www.pondr.com) (Peng et al., 2006).

## Supplementary Material



10.1242/biolopen.062659_sup1 Supplementary information
