# Supplementary material for: Preparation of soluble and functional recombinant HTLV-1 Tax protein using bacterial chaperones
Source: Biol Open. 2026 Jul 3;15(6):bio062659. doi: 10.1242/bio.062659 (PMC13382831; doi:10.1242/bio.062659)
Supplement: Supplementary information [file biolopen-15-062659-s1.pdf]

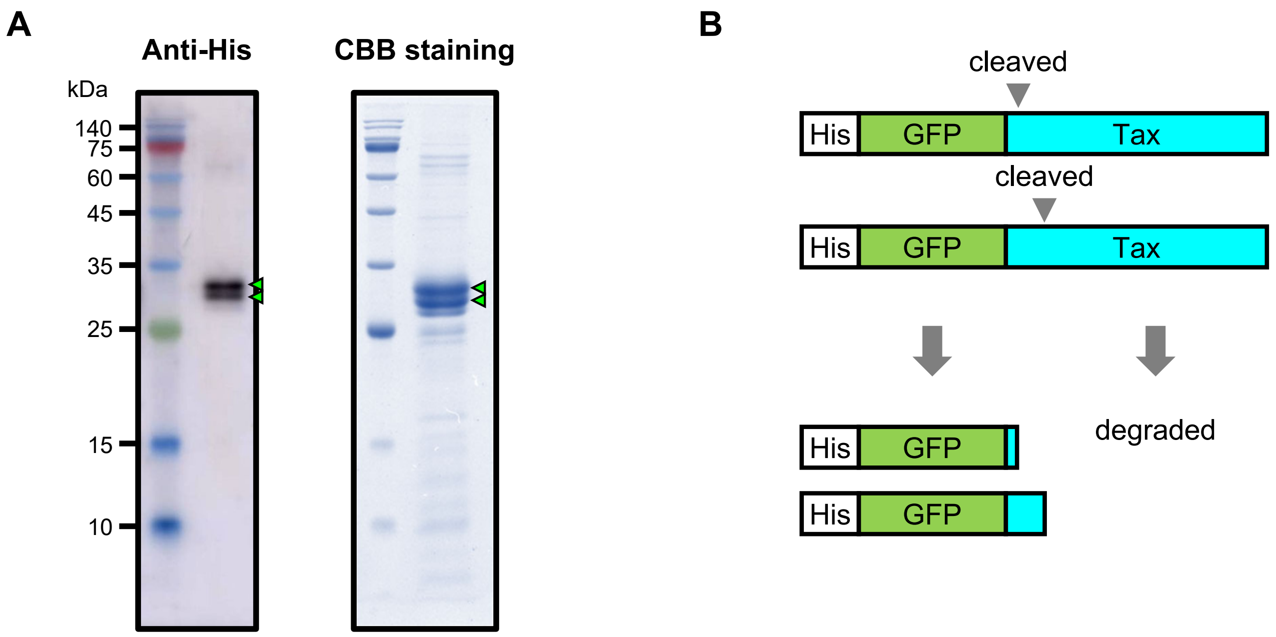

Fig. S1. Western blot with an anti-His antibody to analyse the two major bands detected in Fig. 1B. (A) The Western blot (left panel) reveals that the two major bands detected by CBB-staining (right panel and Fig. 1B) are histidine-tagged. (B) The result suggests that the GFP-fused recombinant Tax protein expressed from the pET vector was cleaved in bacteria, resulting in two fragments containing the intact GFP moiety, whereas Tax in the C-terminal half of the recombinant protein was degraded.

Biology Open • Supplementary information

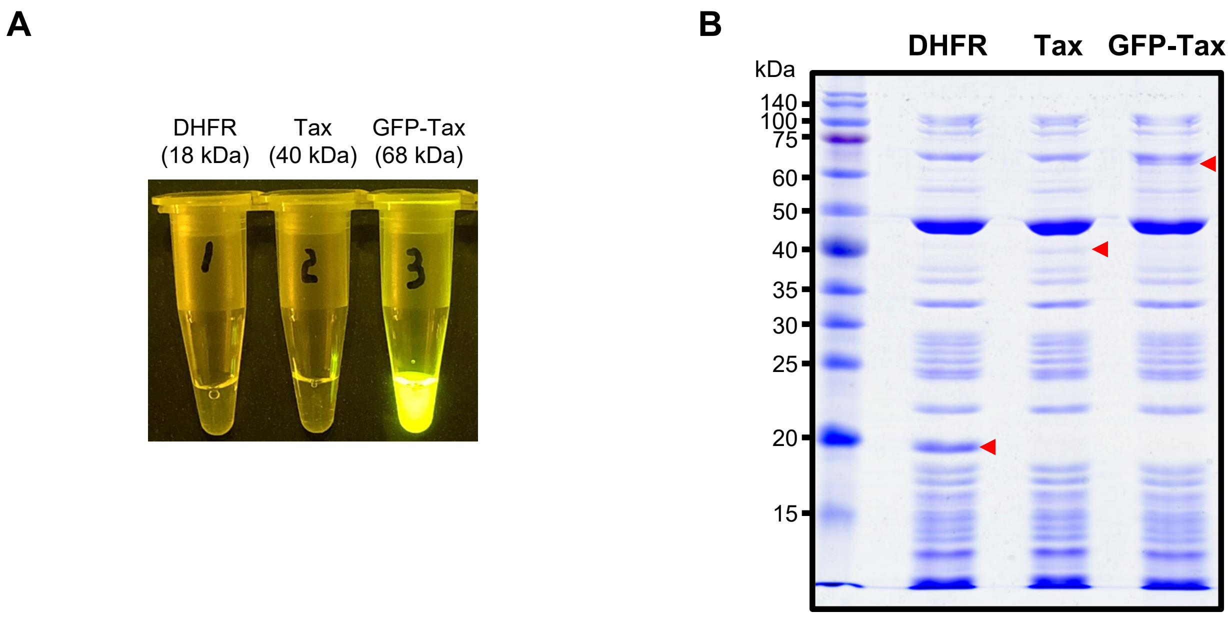

Fig. S2. *In vitro* synthesis of GFP-fused Tax from the pET vector. (A) The reaction was illuminated under a blue LED after the *in vitro* protein synthesis. (B) The proteins in the reaction were resolved by SDS-PAGE. The synthesised proteins are indicated with the red arrowheads.

Biology Open • Supplementary information

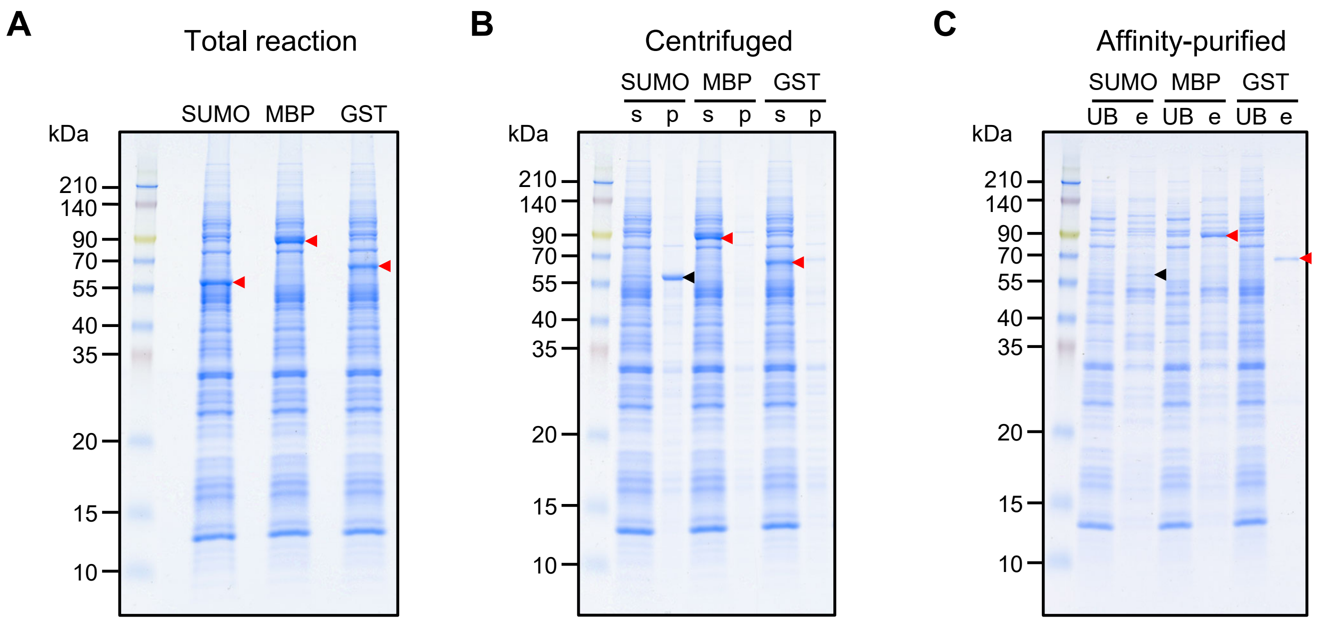

Fig. S3. Testing solubility tags using a wheat germ cell-free expression system. (A) The template plasmid, each encoding HTLV-1 Tax fused to either small ubiquitin-like modifier (SUMO) (52 kDa), maltose-binding protein (MBP) (83 kDa), or glutathione S-transferase (GST) (67 kDa), was transcribed *in vitro*, and the resulting mRNA was translated in the wheat germ extract supplemented with amino acids. Total reaction was resolved by SDS-PAGE. The recombinant protein expressed in each reaction is indicated with a red arrowhead. All three fusion proteins were confirmed for their expression. (B) The total reaction was centrifuged to separate the supernatant from the pellet, and each fraction was resolved by SDS-PAGE (lanes s and p, respectively). The expressed recombinant protein remaining in the supernatant is indicated with a red arrowhead, and the protein found in the pellet with a black arrowhead. MBP-fused and GST-fused recombinant Tax proteins were promising, as they remained in the supernatant when centrifuged to precipitate insoluble material. (C) Unbound and eluted fractions of Ni-NTA (SUMO and MBP) or glutathione resin (GST) purification were resolved by SDS-PAGE. The expressed recombinant protein is marked with an arrowhead in the same colour code as in panel B to indicate successful solubilisation and purification.

Biology Open • Supplementary information

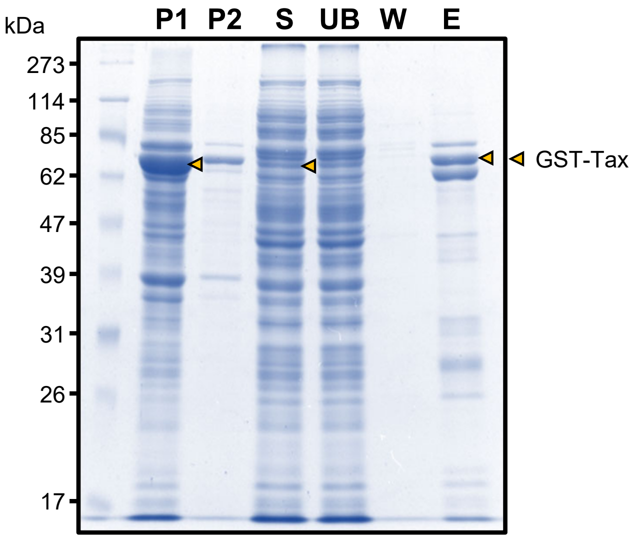

Fig. S4. Expression and purification of GST-fused Tax from the pCold GST vector. The original pCold GST vector, in which Tax was fused to GST, was used to express the recombinant protein in BL21 (DE3) under the cold-shock promoter. The GST-fused Tax (70 kDa) was partially soluble, as evidenced by its presence in the eluate. However, most of the expressed protein precipitated in the bacterial pellet during the centrifugation to clear the lysate. Lanes P1, 2,100×g pellet; P2, 16,000×g pellet; S, supernatant collected after 16,000×g centrifugation; UB, unbound fraction from Ni-NTA; W, 20 mM imidazole wash; E, elution with 400 mM imidazole. The yellow arrowhead indicates the GST-fused Tax.

Biology Open • Supplementary information

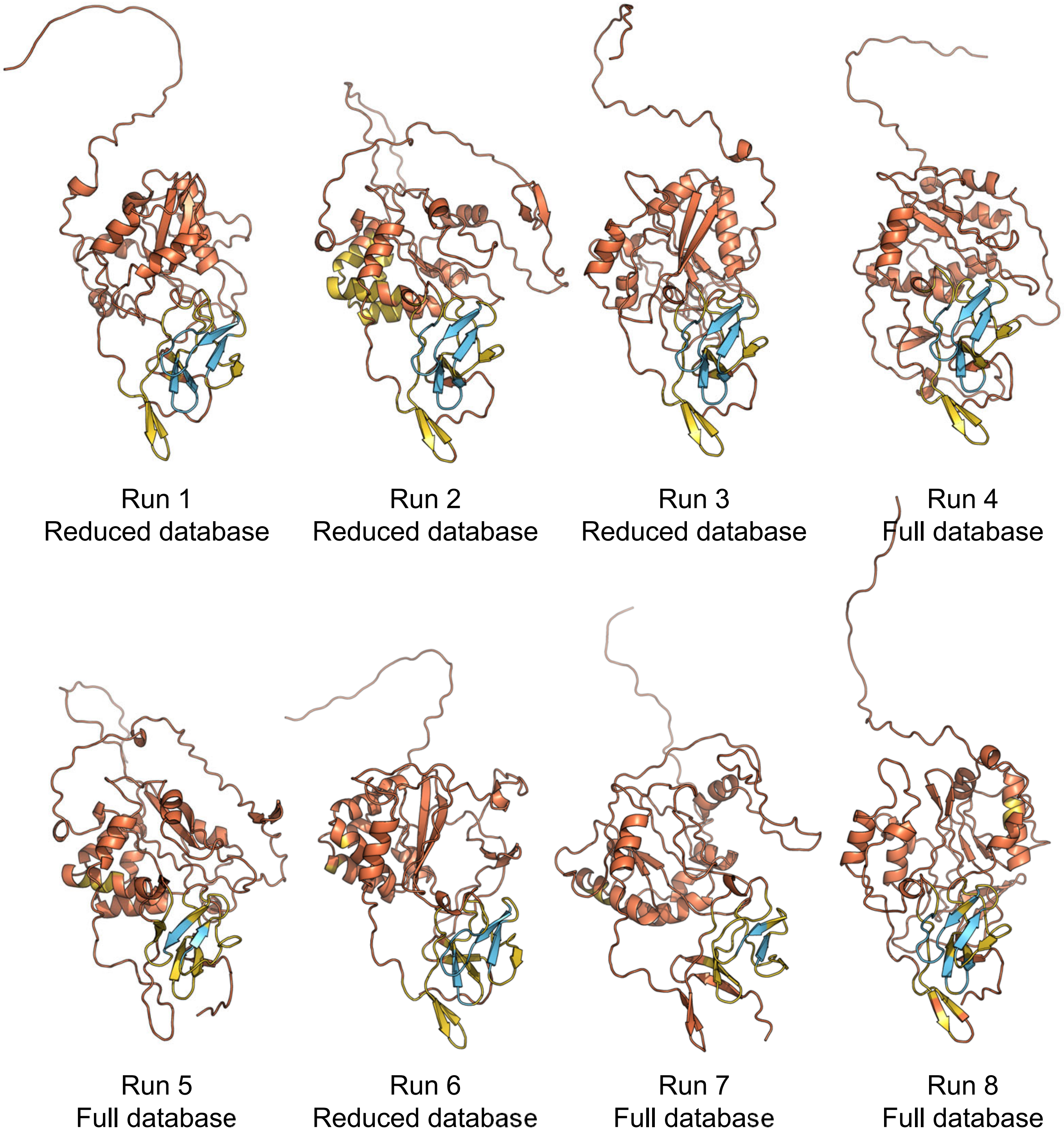

Fig. S5. AlphaFold2 runs on a local computer. Either the full or reduced database was used for prediction, as indicated below the model.

Biology Open • Supplementary information

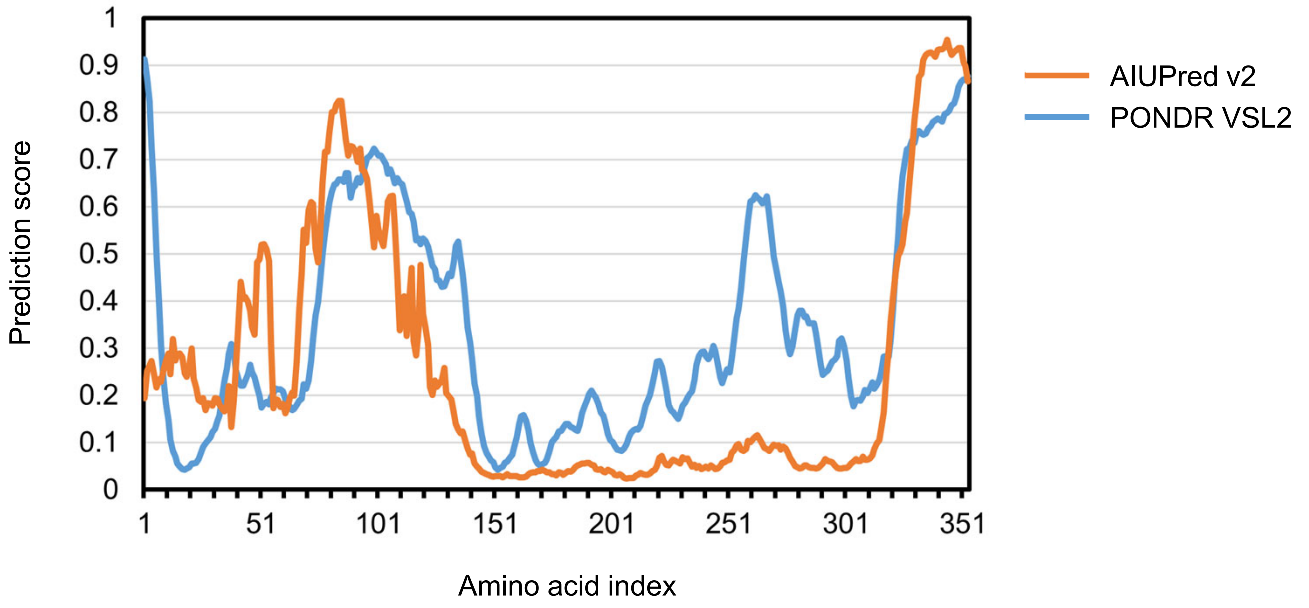

Fig. S6. Prediction of intrinsically disordered regions within HTLV-1 Tax.

Biology Open • Supplementary information

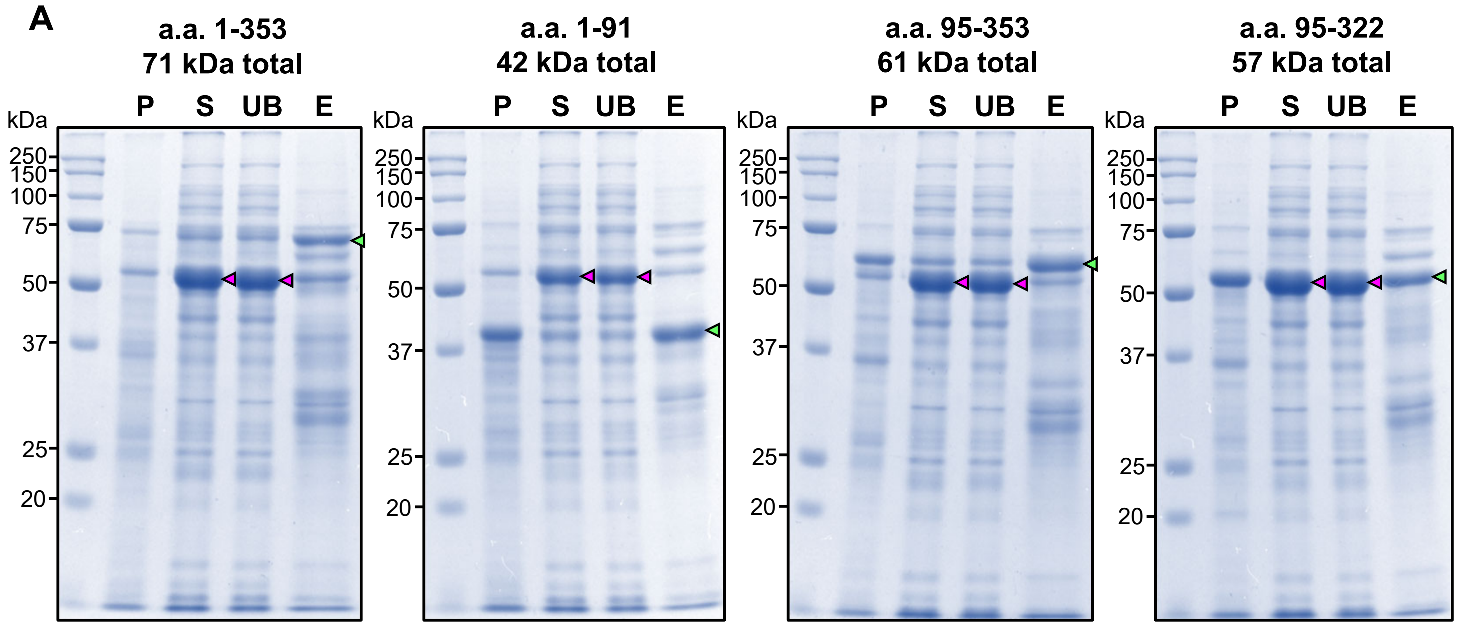

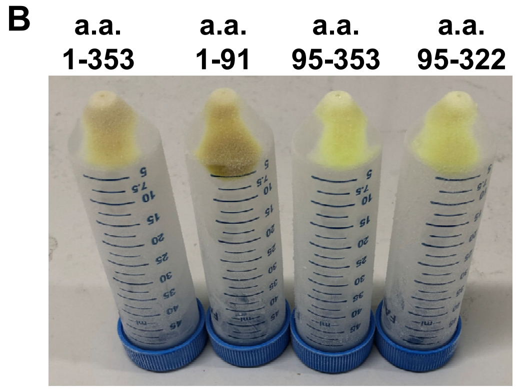

Fig. S7. Expression and purification of GFP-fused Tax from the pCold vector in the presence of exogenous Trigger Factor. (A) Purification of GFP-fused Tax. Lanes P, 2,100×g pellet; S, supernatant collected after 16,000×g centrifugation; UB, unbound fraction of Ni-NTA; E, elution. The green arrowhead indicates the expressed recombinant protein, and magenta indicates Trigger Factor. Molecular weight of the total recombinant protein is indicated above each panel. (B) The bacteria pellet harvested at the bottom of the tube after the overnight induction of the recombinant protein.

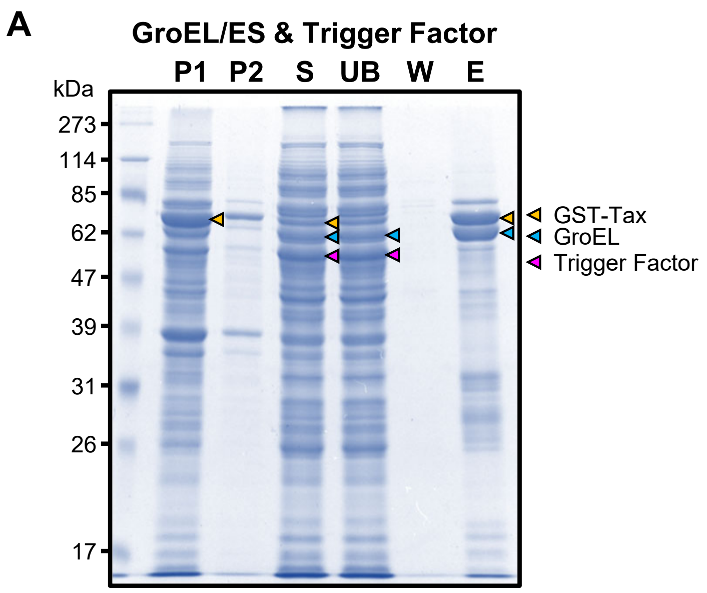

Fig. S8. The effect of bacterial chaperones. (A) Purification of GST-fused Tax expressed with GroEL/ES and Trigger Factor. Lanes P1, 2,100×g pellet; P2, 16,000×g pellet; S, supernatant collected after 16,000×g centrifugation; UB, unbound fraction from Ni-NTA; W, 20 mM imidazole wash; E, elution with 400 mM imidazole. The yellow arrowhead is for GST-fused Tax, magenta for Trigger Factor, and blue for GroEL, judged by the molecular weight. The GroEL/GroES chaperones, together with Trigger Factor, slightly improved the solubility of the GST-fused recombinant Tax protein. However, GroEL was found co-purified with Tax. (B) Purification of GST-fused Tax expressed with GroEL/ES and DnaKJE. The lane indication is the same as in panel A. The yellow arrowhead is for GST-fused Tax, blue for GroEL, and green for DnaJ, judged by the molecular weight. GroEL and DnaJ were co-purified with the recombinant Tax protein.

Biology Open • Supplementary information

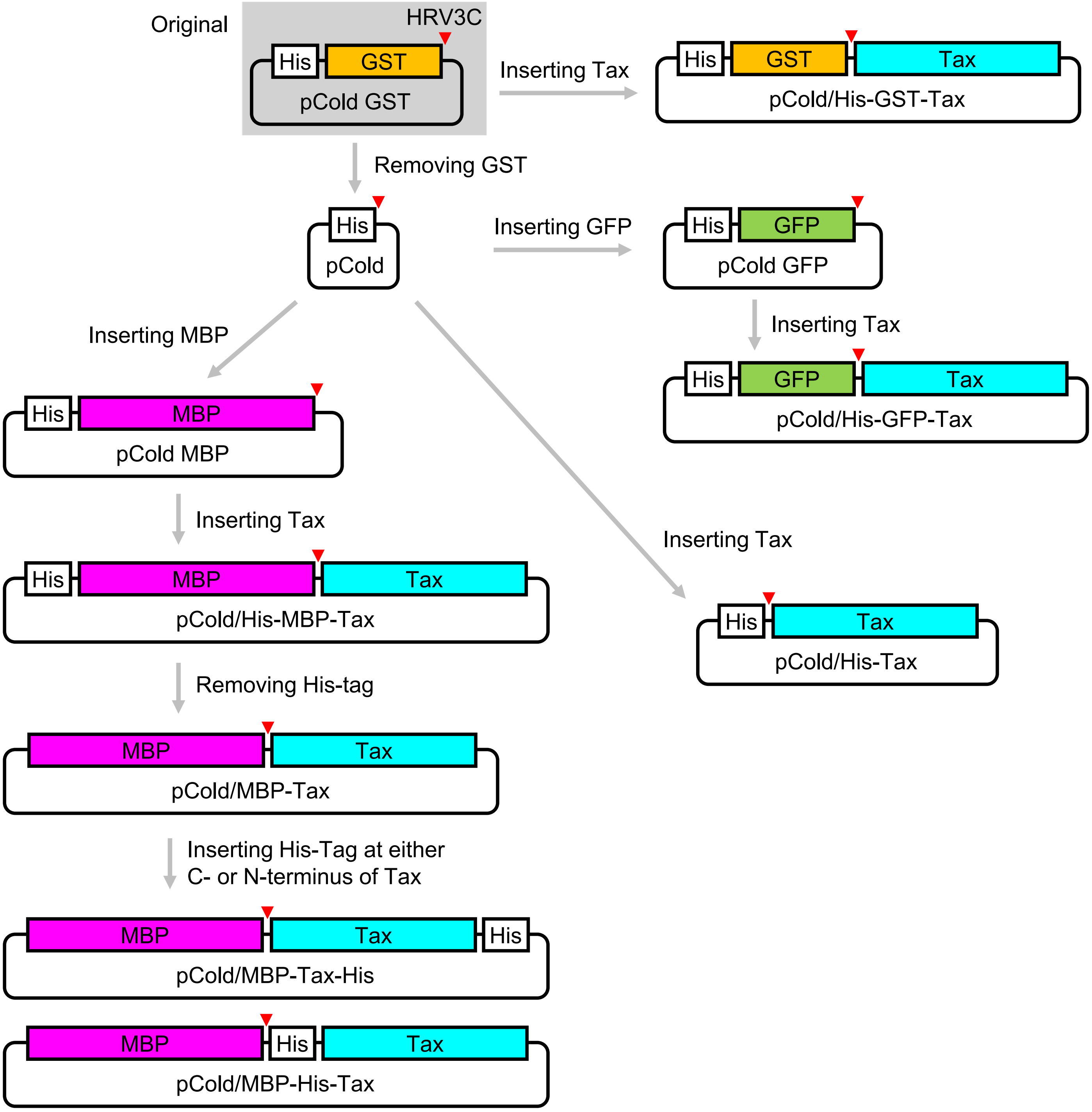

Fig. S9. Schematic diagrams of pCold expression plasmids constructed and used in this study.

Biology Open • Supplementary information
