# Supplementary material for: Preparation of soluble and functional recombinant HTLV-1 Tax protein using bacterial chaperones
Source: Biol Open. 2026 Jul 3;15(6):bio062659. doi: 10.1242/bio.062659 (PMC13382831; doi:10.1242/bio.062659)
Supplement: Supplementary file 1 [file biolopen-15-062659_review_history.pdf]

Preparation of soluble and functional recombinant HTLV-1 Tax protein using bacterial chaperones

Michi Miura, Kazutaka Murayama, Satoshi Uemura, Hiroshi Kitamura, Mineki Saito and Teru Kanda

10.1242/bio.062659

Editor: Christopher A. Maher

Review timeline

Original submission:	28 April 2026
Editorial decision:	30 April 2026
First revision received:	14 May 2026
Accepted:	18 May 2026

Original submission

First decision letter

MS ID#: bio.062659

MS Title: Preparation of soluble and functional recombinant HTLV-1 Tax protein using bacterial chaperones

Authors: Michi Miura, Kazutaka Murayama, Satoshi Uemura, Hiroshi Kitamura, Mineki Saito and Teru Kanda

I have now reached a decision on the above manuscript.

Your manuscript was reviewed as part of our Fast & Fair peer review initiative, an experiment to offer authors decisions, with high quality reviews, within 7 working days of initial submission. This approach uses contracted reviewers (paid 220 GBP per manuscript) who agree to complete high-quality reviews within a set timeframe. Our goal is to minimise delays associated with peer review while maintaining the highest standards of rigor and integrity. To learn more, read the Editorial by Daniel Gorelick, or visit the Fast & Fair peer review page. If you have any feedback on our initiative, we'd love to hear from you fast.fair@biologists.com

The reviewer reports are shown at the bottom of this email.

As you will see, the reviewers gave favourable reports, but raised some critical points that will require amendments to your manuscript. I hope that you will be able to carry these out, because we would like to be able to accept your paper.

At this stage, we also ask you to ensure your manuscript complies with our formatting guidelines - please see our manuscript preparation guidelines for details. Provided you are able to fully address the referees' comments, we are positive about publication of your paper (we accept over 95% of revision submissions) and therefore hope you won't mind any extra work involved in reformatting your manuscript at this point.

Please upload both a 'clean' version of your Word file, along with a highlighted version clearly showing where you have made changes in the revised manuscript. Please avoid using 'Track changes' in Word files as these are lost in PDF conversion.

© 2026. Published by The Company of Biologists under the terms of the Creative Commons Attribution License (https://creativecommons.org/licenses/by/4.0/).

I should be grateful if you would also provide a point-by-point response detailing how you have dealt with the points raised by the reviewers in the 'Response to Reviewers' box. Please attend to all of the reviewers' comments. If you do not agree with any of their criticisms or suggestions please explain clearly why this is so.

Reviewer 1

Comments for the author

In this nicely written manuscript, Miura et al. aimed to disseminate the methods for preparation of soluble, functional recombinant full length and truncated HTLV-1 Tax protein using bacterial chaperones.
It is indeed a prerequisite for the biochemical characterization of this enigmatic and challenging protein from HTLV-1 virus.
I truly appreciated the meticulous work and description of observations during the protein purification.

Only two important comments:
-Please provide a step-by-step protocol on the Tax protein preparation as a supplementary material file.
- Please share the plasmids in an open repository (such as addgene).

Minor comments:
1) Please add a brief biochemical description of Tax protein in the introduction: aa length and kDa.
2) Please add the molecular mass (kDa) of the different polyprotein constructs whenever they are introduced in the results section.
3) Please add a label in all SDS-PAGE to indicate that the values of markers (the protein ladder) correspond to "kDa".
4) Please further discuss alternative explanations to this observation (Fig1A-B):
a. Line 106-107 "These results suggest that Tax was unstable and degraded in bacteria, leaving only the GFP moiety intact"
b. Line 235-236 " the Tax moiety was completely degraded in bacteria"
My questions are: Is it protein degradation or cleavage? do you have evidence of protein degradation (vs cleavage)?
5) Line 251: "Tax is potentially a heme-binding protein".
Remarkable observation!! and thanks for further narrowing down the investigation to the first 90 aa. I'm looking forward to reading the follow up biochemical investigation into the structure and function of this domain.
6) Line 314-315: "we aimed to disseminate the preparation of soluble, functional recombinant HTLV-1 Tax using the bacterial chaperones".
This statement is more suitable for the introduction (than the discussion). Although a similar statement is already there, rendering this statement in the discussion mostly redundant.

Reviewer 2

Comments for the author

This manuscript addresses a long standing technical bottleneck in the HTLV 1 field, the difficulty of producing soluble, functional recombinant Tax protein for biochemical and structural studies. The authors systematically evaluate solubility tags and bacterial chaperones and demonstrate that co expression with Trigger Factor, particularly in combination with MBP, enables purification of full length and truncated Tax proteins that retain NF κB-activating activity. The work is primarily methodological but is strengthened by functional validation and complementary in silico analyses. Overall, the study is technically sound, clearly written, and of tangible value to the field. Several points regarding quantitative rigor, reproducibility, and interpretation should be addressed to strengthen the manuscript.

1. Experimental quality

© 2026. Published by The Company of Biologists under the terms of the Creative Commons Attribution License (https://creativecommons.org/licenses/by/4.0/).

The solubility and purification experiments include appropriate fractionation controls, which clearly demonstrate solubility improvements. The NF κB activation assay includes a well chosen negative control (Tax M22 mutant), which strongly supports the claim that activity is intrinsic to the recombinant protein rather than bacterial contaminants. Loading controls are appropriate and adequately shown. The use of solubility tags (His, GST, MBP), low temperature expression, and bacterial chaperones is well justified for intrinsically disordered proteins. Trigger Factor is an appropriate and mechanistically plausible choice, and the side by side comparison with other chaperones (Supplementary data) strengthens this conclusion. The in vitro NF κB activation assay is an appropriate functional readout for Tax's canonical activity and fits the stated objective of demonstrating functionality rather than full physiological recapitulation.

Statistical analysis
Most experiments are qualitative or semi quantitative (SDS PAGE, western blots). No statistical analyses are presented for the NF κB assay or solubility comparisons. While this is common for method development studies, the authors should clarify how many independent purifications and activations were performed, and whether the shown blots are representative.

2. Reproducibility
The manuscript does not consistently specify the number of independent expression/purification experiments performed for each construct. The NF κB assay appears to be shown from a single experiment; it is unclear whether the result was reproduced independently. Fractionation gels and blots are shown clearly, but quantitative raw data are not provided. The authors state that original images and PDB coordinates are available upon request, which is acceptable but could be improved by explicit deposition where feasible. The Methods section is detailed and thorough, with sufficient information on plasmids, strains, induction conditions, purification buffers, and simulation parameters to permit reproduction. Domain cloning strategies and primer sequences are clearly provided.

3. Completeness
The main conclusions that Trigger Factor enables soluble expression of Tax, that the purified protein is functional, and that predicted domains can be prepared are supported by the data. Claims are generally conservative and appropriately framed as enabling future structural studies rather than delivering definitive structural insights.

Potential design flaws
No major experimental design flaws are apparent. One limitation is that functional validation is limited to NF κB activation; other known Tax activities (e.g., CREB interaction) are not tested. Demonstration that Tax activity is retained after complete removal of MBP (e.g., SEC purified Tax alone) would further strengthen the conclusions. The heme binding observation is intriguing but remains speculative without direct biochemical evidence. The Discussion appropriately notes the challenges of crystallography, limitations of NMR for full length Tax, and the need for further purification steps for structural work. The speculative nature of the heme binding claim is acknowledged implicitly but could be stated more explicitly.

4. Scholarship
The manuscript cites seminal and recent literature on intrinsically disordered proteins, HTLV 1 biology, and bacterial chaperones. Prior reports of Tax insolubility and bacterial expression difficulties are appropriately discussed. The authors cite studies that highlight Tax's promiscuous interactions and disordered nature, which support their rationale. There is no significant omission of relevant contradictory literature; the work does not directly challenge existing models but rather fills a technical gap. The authors clearly describe the state of the field and explain how their approach improves upon previous attempts by combining Trigger Factor with MBP and a practical starch based purification method. The novelty is appropriately framed as methodological rather than conceptual.

Overall recommendation

The manuscript is of good quality and will be valuable to researchers studying HTLV 1 Tax and intrinsically disordered viral proteins. Addressing my above raised points would significantly

© 2026. Published by The Company of Biologists under the terms of the Creative Commons Attribution License (https://creativecommons.org/licenses/by/4.0/).

strengthen rigor and clarity. With these revisions, the manuscript will be suitable for publication.

Reviewer's Responses to Questions

Experimental quality
Are experiments performed using appropriate methods that will answer the question (or test the hypothesis or support the observations) posed by the authors? Is the right tool used for the job?
If 'No', please indicate reasons in Comments for Author box below.
Reviewer #1:
- Yes

Reviewer #2:
- Yes

Were the data analyzed using appropriate statistical tests?
If 'No', please indicate reasons in Comments for Author box below.
Reviewer #1:
- Yes

Reviewer #2:
- Yes

Reproducibility
Were experiments performed using adequate number of biological replicates?
If 'No', please indicate reasons in Comments for Author box below.
Reviewer #1:
- Yes

Reviewer #2:
- Yes

Is there sufficient raw data to assess the rigor of the analysis?
If 'No', please indicate reasons in Comments for Author box below.
Reviewer #1:
- Yes

Reviewer #2:
- Yes

Does the methods section provide sufficient detail to permit reproducibility?
If 'No', please indicate reasons in Comments for Author box below.
Reviewer #1:
- Yes

Reviewer #2:
- Yes

Completeness
Are the manuscript's conclusions supported by the data?
If 'No', please indicate reasons in Comments for Author box below.
Reviewer #1:
- Yes

Reviewer #2:

© 2026. Published by The Company of Biologists under the terms of the Creative Commons Attribution License (https://creativecommons.org/licenses/by/4.0/).

- Yes

Scholarship
Do the authors cite and discuss the merits of data that would argue for and against their conclusion?
If 'No', please indicate reasons in Comments for Author box below.
Reviewer #1:
- Yes

Reviewer #2:
- Yes

Does the manuscript title & abstract accurately reflect the contents of the manuscript, without hyperbole?
If 'No', please indicate reasons in Comments for Author box below.
Reviewer #1:
- Yes

Reviewer #2:
- Yes

First revision

Author response to reviewers' comments

Point-by-point response to the reviewers' comments

We thank the reviewers for their thoughtful and constructive feedback. We have addressed each comment below and made the necessary alterations to the main text and figures that we highlighted to facilitate the review.

Reviewer 1: Summary:

In this nicely written manuscript, Miura et al. aimed to disseminate the methods for preparation of soluble, functional recombinant full length and truncated HTLV-1 Tax protein using bacterial chaperones.

It is indeed a prerequisite for the biochemical characterization of this enigmatic and challenging protein from HTLV-1 virus.

I truly appreciated the meticulous work and description of observations during the protein purification.

Only two important comments:

-Please provide a step-by-step protocol on the Tax protein preparation as a supplementary material file.
Response 1-1: Instead of describing the method in a supplementary file that does not comply with the journal policy, we posted the protocol on protocols.io, a popular online platform for sharing detailed, step-by-step protocols at https://www.protocols.io/view/purification-of-mbp-fused-htlv-1-tax-miura-et-al-2-kxygxj6n4l8j/v1. The Materials and Methods section has been updated accordingly (lines 414-416).

- Please share the plasmids in an open repository (such as addgene).

© 2026. Published by The Company of Biologists under the terms of the Creative Commons Attribution License (https://creativecommons.org/licenses/by/4.0/).

Response 1-2: We do understand the convenience of sharing the plasmids via Addgene. However, the plasmids we used were derived from a commercial source under license, and we are not certain if we are permitted to deposit the modified plasmids to Addgene. Therefore, upon receiving a request to provide our plasmids, we will do so by liaising with our MTA office, the destination institution, and the commercial company to ensure we do not breach any licensing terms.

Minor comments:

1) Please add a brief biochemical description of Tax protein in the introduction: aa length and kDa.

Response 1-3: Added the description on Tax in lines 54-55: "HTLV-1 Tax, originally referred to as p40 (Sugamura et al., 1984), is one of these viral proteins comprising 353 amino acids, and is responsible for viral pathogenesis."

2) Please add the molecular mass (kDa) of the different polyprotein constructs whenever they are introduced in the results section.

Response 1-4: Added the following:
- His-Tax from pET, 40 kDa (line 97)
- GFP-Tax from pET, 68 kDa (line 102, and the molecular mass of GFP-Tax in Fig. S2A was corrected.)
- SUMO-Tax, 52 kDa; MBP-Tax, 83 kDa; and GST-Tax, 67 kDa for wheat-germ expression (Fig. S3)
- GST-Tax from pCold, 70 kDa (Fig. S4 and line 129)
- MBP-Tax, 86 kDa (line 135); and MBP-fused Tax fragments, 56 kDa, 76 kDa, 72 kDa (line 228)
- GFP-fused Tax from pCold, 71 kDa, 42 kDa, 61 kDa, 57 kDa (Fig. S7A)
- His-Tax from pCold, 44 kDa, 15 kDa, 34 kDa, 30 kDa (Fig. 5B).

In addition, the molecular weight of the MBP moiety (45 kDa) and Tax (full-length, 41 kDa, and three truncated fragments, 11 kDa, 31 kDa, and 27 kDa) were added (line 170 and Fig. 5A). Figure legends have been updated accordingly (lines 671-672, 675-676, and Fig. S7).

3) Please add a label in all SDS-PAGE to indicate that the values of markers (the protein ladder) correspond to "kDa".

Response 1-5: Added. Thank you for raising this important point.

4) Please further discuss alternative explanations to this observation (Fig1A-B):
a. Line 106-107 "These results suggest that Tax was unstable and degraded in bacteria, leaving only the GFP moiety intact"
b. Line 235-236 " the Tax moiety was completely degraded in bacteria"

My questions are: Is it protein degradation or cleavage? do you have evidence of protein degradation (vs cleavage)?

Response 1-6: We have performed an additional Western blot (now Fig. S1) to confirm that the major bands detected in Fig. 1B are histidine-tagged N-terminal GFP. This additional work provides evidence that Tax in the C-terminal half of the recombinant protein was degraded. The new Western blot figure has been added (Fig. S1), the Result section has been updated (lines 105-108), and the label on Fig. 1B indicating the protein bands has been changed to "GFP fragments". We have removed "completely" from line 241, as we are not certain to what extent Tax was degraded.

5) Line 251: "Tax is potentially a heme-binding protein".

Remarkable observation!! and thanks for further narrowing down the investigation to the first 90 aa. I'm looking forward to reading the follow up biochemical investigation into the structure and function of this domain.

© 2026. Published by The Company of Biologists under the terms of the Creative Commons Attribution License (https://creativecommons.org/licenses/by/4.0/).

Response 1-7: Thank you for your interest. We are performing spectrophotometry and MALDI mass spec, among other things, for further biochemical investigation.

6) Line 314-315: "we aimed to disseminate the preparation of soluble, functional recombinant HTLV-1 Tax using the bacterial chaperones".

This statement is more suitable for the introduction (than the discussion). Although a similar statement is already there, rendering this statement in the discussion mostly redundant.

Response 1-8: We have made an alteration in line 318 to make it sound more like a conclusion: "In this paper, we demonstrated the preparation of soluble, functional recombinant HTLV-1 Tax using the bacterial chaperones. Additional purification, such as size-exclusion chromatography, is required to improve the purity of the recombinant proteins for structural studies. Nevertheless, we believe that this work takes the first step towards the structural characterisation of HTLV-1 Tax."

Reviewer 2: This manuscript addresses a long standing technical bottleneck in the HTLV 1 field, the difficulty of producing soluble, functional recombinant Tax protein for biochemical and structural studies. The authors systematically evaluate solubility tags and bacterial chaperones and demonstrate that co expression with Trigger Factor, particularly in combination with MBP, enables purification of full length and truncated Tax proteins that retain NF κB-activating activity. The work is primarily methodological but is strengthened by functional validation and complementary in silico analyses. Overall, the study is technically sound, clearly written, and of tangible value to the field. Several points regarding quantitative rigor, reproducibility, and interpretation should be addressed to strengthen the manuscript.

1. Experimental quality

The solubility and purification experiments include appropriate fractionation controls, which clearly demonstrate solubility improvements. The NF κB activation assay includes a well chosen negative control (Tax M22 mutant), which strongly supports the claim that activity is intrinsic to the recombinant protein rather than bacterial contaminants. Loading controls are appropriate and adequately shown. The use of solubility tags (His, GST, MBP), low temperature expression, and bacterial chaperones is well justified for intrinsically disordered proteins. Trigger Factor is an appropriate and mechanistically plausible choice, and the side by side comparison with other chaperones (Supplementary data) strengthens this conclusion. The in vitro NF κB activation assay is an appropriate functional readout for Tax's canonical activity and fits the stated objective of demonstrating functionality rather than full physiological recapitulation.

Statistical analysis

Most experiments are qualitative or semi quantitative (SDS PAGE, western blots). No statistical analyses are presented for the NF κB assay or solubility comparisons. While this is common for method development studies, *the authors should clarify how many independent purifications and activations were performed, and whether the shown blots are representative.*

Response 2-1: We have updated the figure legends (lines 621-622, 636-637, 645-646, 678-679). Purifications were performed at least twice, and the NF-kappaB activation assay was performed twice using independent protein preparations to confirm reproducibility.

2. Reproducibility

The manuscript does not consistently specify the number of independent expression/purification experiments performed for each construct. The NF κB assay appears to be shown from a single experiment; it is unclear whether the result was reproduced independently. Fractionation gels and blots are shown clearly, but quantitative raw data are not provided. The authors state that original images and PDB coordinates are available upon request, which is acceptable but could be improved by explicit deposition where feasible. The Methods section is detailed and thorough, with sufficient information on plasmids, strains, induction conditions, purification buffers, and simulation parameters to permit reproduction. Domain cloning strategies and primer sequences are clearly provided.

© 2026. Published by The Company of Biologists under the terms of the Creative Commons Attribution License (https://creativecommons.org/licenses/by/4.0/).

Response 2-2: For reproducibility, please see our Response 2-1. For data availability, we have uploaded the original images of CBB-stained gels and Western blots, and the PDB coordinates of AlphaFold predictions and the molecular dynamics simulation on https://osf.io/bwef6/files/osfstorage. The Data availability statement was updated accordingly (lines 487-489).

3. Completeness

The main conclusions that Trigger Factor enables soluble expression of Tax, that the purified protein is functional, and that predicted domains can be prepared are supported by the data. Claims are generally conservative and appropriately framed as enabling future structural studies rather than delivering definitive structural insights.

Potential design flaws

No major experimental design flaws are apparent. One limitation is that functional validation is limited to NF κB activation; other known Tax activities (e.g., CREB interaction) are not tested. Demonstration that Tax activity is retained after complete removal of MBP (e.g., SEC purified Tax alone) would further strengthen the conclusions. The heme binding observation is intriguing but remains speculative without direct biochemical evidence. The Discussion appropriately notes the challenges of crystallography, limitations of NMR for full length Tax, and the need for further purification steps for structural work. *The speculative nature of the heme binding claim is acknowledged implicitly but could be stated more explicitly.*

Response 2-3: Regarding the heme-binding, we have rephrased in line 265: “These observations raise the possibility that Tax binds heme primarily within the first 90 amino acids, and that heme binding accounts for the brownish appearance of Tax.”

4. Scholarship

The manuscript cites seminal and recent literature on intrinsically disordered proteins, HTLV 1 biology, and bacterial chaperones. Prior reports of Tax insolubility and bacterial expression difficulties are appropriately discussed. The authors cite studies that highlight Tax's promiscuous interactions and disordered nature, which support their rationale. There is no significant omission of relevant contradictory literature; the work does not directly challenge existing models but rather fills a technical gap. The authors clearly describe the state of the field and explain how their approach improves upon previous attempts by combining Trigger Factor with MBP and a practical starch based purification method. The novelty is appropriately framed as methodological rather than conceptual.

Overall recommendation

The manuscript is of good quality and will be valuable to researchers studying HTLV 1 Tax and intrinsically disordered viral proteins. Addressing my above raised points would significantly strengthen rigor and clarity. With these revisions, the manuscript will be suitable for publication.

Second decision letter

MS ID#: bio.062659R1

MS Title: Preparation of soluble and functional recombinant HTLV-1 Tax protein using bacterial chaperones

Authors: Michi Miura, Kazutaka Murayama, Satoshi Uemura, Hiroshi Kitamura, Mineki Saito and Teru Kanda

© 2026. Published by The Company of Biologists under the terms of the Creative Commons Attribution License (https://creativecommons.org/licenses/by/4.0/).

I am happy to tell you that your manuscript has been accepted for publication in Biology Open, pending our standard publication integrity checks. It was accepted on 18th May 2026.

© 2026. Published by The Company of Biologists under the terms of the Creative Commons Attribution License (https://creativecommons.org/licenses/by/4.0/).
